# Transition metal dyshomeostasis-associated macrophage dysfunction: potential roles in chronic metabolic diseases

**DOI:** 10.3389/fimmu.2026.1950688

**Published:** 2026-10-27

**Authors:** Bixian Sun, Jinhan Yang, Yuanyuan Shen, Runyi Liu, Mingyue Ma, Yue Wang

**Affiliations:** 1 The First Clinical Medical College, Shenyang Medical College, Shenyang, China; 2 Department of Forensic Medicine, Criminal Investigation Police University of China, Shenyang, China; 3 School of Public Health, Shenyang Medical College, Shenyang, China; 4 Liaoning Key Laboratory of Environmental Pollution and Microecology, Shenyang Medical College, Shenyang, China

**Keywords:** iron, macrophage polarization, metabolic diseases, metal homeostasis, zinc

## Abstract

Transition metals such as iron, zinc, and copper serve as indispensable cofactors in immunometabolic regulation. As central regulators of innate immunity, macrophages rely on strict metal homeostasis to maintain their functional plasticity. Studies have shown that transition metal dyshomeostasis is associated with aberrant macrophage polarization and sustained inflammatory responses. Such macrophage dysfunction may contribute to the initiation and progression of chronic systemic metabolic diseases. This review comprehensively summarizes the regulatory functions of essential transition metals in shaping macrophage inflammatory phenotypes and regulating metabolic reprogramming. We further clarify the molecular mechanisms by which metal-induced macrophage dysfunction participates in the pathogenesis of major metabolic diseases such as type 2 diabetes mellitus, metabolic dysfunction-associated steatotic liver disease, and atherosclerosis. We discuss how targeting intracellular metal homeostasis via selective metal chelators and novel metal-based nanomaterials represents a promising and innovative immunomodulatory therapeutic strategy. Essential transition metal homeostasis is a critical determinant of macrophage plasticity and appears to play a significant role in the pathogenesis of chronic metabolic diseases. Macrophage dysfunction induced by transition metal dyshomeostasis is associated with systemic insulin resistance and may contribute to tissue-specific damage within hepatic, vascular, and adipose microenvironments. Although the mechanistic foundations have been established, significant knowledge gaps remain regarding stage-specific variations and dose-dependent thresholds of metal-induced macrophage phenotypic shifts. Future research should prioritize the validation of metal-based biomarkers in clinical cohorts and the development of targeted ‘metal-homeostasis’ therapies to restore macrophage function, thereby providing a novel theoretical framework for the prevention and treatment of chronic metabolic disorders.

## Highlights

- Metal imbalance alters macrophage polarization via NF-κB, HIF-1α, and cGAS/STING.
- Fe, Zn, Cu, Co, Mn regulate macrophage function in MASLD/NAFLD, T2DM, and AS.
- Metal-targeted therapies show preclinical potential to ameliorate macrophage dysfunction and modulate immunity; clinical efficacy remains to be established.

## 1 Introduction

Essential transition metals, including iron, zinc, copper, cobalt, and manganese, are indispensable trace elements that regulate immune cell development, proliferation, and signal transduction of immune cells (1–3). As core innate immune cells, macrophages are sensitive to transition metal homeostasis. Iron and manganese regulate macrophage polarization; copper oxide nanoparticles may trigger NLRP3 inflammasome activation and pro-inflammatory mediator release, and zinc deficiency impairs macrophage-mediated inflammatory responses (4–6). Maintaining homeostasis of these transition metals is therefore critical for preserving macrophage function and immune homeostasis.

Metabolic diseases including type 2 diabetes mellitus (T2DM), metabolic dysfunction-associated steatotic liver disease (MASLD), and atherosclerosis (AS) are now recognized as immunometabolic disorders centrally involving macrophage dysfunction. Macrophage dysfunction is implicated in inflammation-driven metabolic disturbances, including hepatic lipid accumulation, foam cell formation, and pancreatic β-cell damage (7–9). Accumulating evidence suggests that transition metals may influence the onset and progression of these metabolic diseases by modulating macrophage function (9–11). However, the shared and metal-specific impacts of key transition metals on macrophage immunometabolism are not systematically understood, particularly their divergent roles in distinct metabolic diseases. The signaling mechanisms linking metal dyshomeostasis to macrophage dysfunction remain poorly characterized, and an integrated model is needed to describe context-dependent macrophage phenotypic shaping by perturbed metal homeostasis in chronic disease microenvironments.

Clarifying the crosstalk among transition metal homeostasis, macrophage function, and chronic disease pathogenesis is essential for identifying novel therapeutic targets. This review highlights how iron, zinc, copper, cobalt and manganese modulate macrophage function, and how disrupted metal homeostasis may contribute to macrophage dysfunction to potentially drive chronic metabolic diseases. Transition metals and macrophages interact bidirectionally: inflammatory/metabolic signals alter metal transporter handling and remodel macrophage phenotypes. These interactions form metal–macrophage immunometabolic feedback loops that may sustain chronic inflammation and metabolic impairment.

## 2 Dependence of macrophage function on essential transition metals

### 2.1 Macrophages: core roles in immunity and chronic disease pathogenesis

As a primary component of host defense, the innate immune system provides the first line of response to pathogenic invasion and relies on physical barriers, soluble mediators, and specialized immune cells such as macrophages, neutrophils, and dendritic cells, to mount rapid, non-specific immune responses (12). As central effector cells of the innate immune system, macrophages perform diverse and essential functions, including pathogen recognition, phagocytosis, regulation of inflammation, and maintenance of tissue homeostasis. They recognize pathogen-associated molecular patterns (PAMPs) through pattern-recognition receptors (PRRs), such as Toll-like receptors (TLRs), thereby activating downstream signaling pathways that induce antimicrobial gene expression and inflammatory cytokine secretion. In addition, macrophages eliminate pathogens and damaged cells through phagocytosis and lysosomal degradation (12).

Macrophages exhibit remarkable phenotypic plasticity, enabling them to adopt distinct functional states in response to environmental cues. These states include the resting M0 phenotype, the classically activated pro-inflammatory M1 phenotype, and the alternatively activated anti-inflammatory M2 phenotype. M1 macrophages, which are induced by stimuli such as lipopolysaccharide (LPS) and interferon-γ (IFN-γ), produce pro-inflammatory cytokines, including tumor necrosis factor-α (TNF-α), interleukin-1β (IL-1β), and interleukin-6 (IL-6). By contrast, M2 macrophages, which are driven by interleukin-4 (IL-4) and interleukin-13 (IL-13), secrete anti-inflammatory cytokines such as interleukin-10 (IL-10) and contribute to tissue repair (2, 13, 14). In addition, TLR activation in macrophages enhances antigen-presenting capacity and promotes dendritic cell maturation as well as the upregulation of costimulatory molecules, thereby linking innate and adaptive immunity (12). Accordingly, **Figure 1** provides a schematic overview of macrophage polarization and its functional outputs.

**Figure 1:**
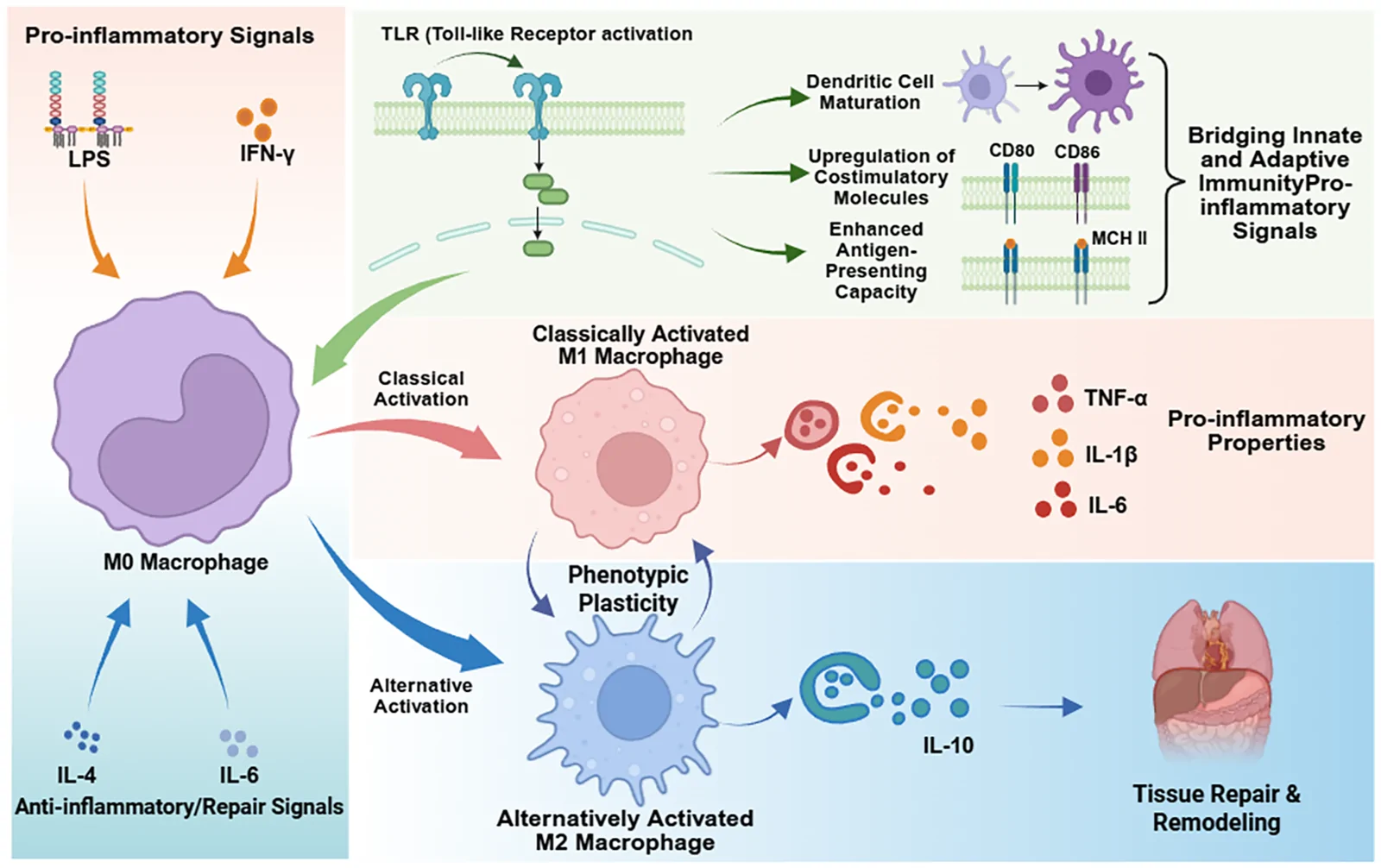
Phenotypic plasticity of macrophages and their functional roles in innate immunity and tissue homeostasis. Caption: M1/M2 labels denote simplified experimental activation programs and do not represent exhaustive in vivo macrophage categories. Tissue origin, local niche and disease stage jointly shape macrophage states and functional outcomes. Schematic was created with [BioRender.com] (https://BioRender.com).

Importantly, macrophage dysfunction is a common pathological feature of chronic metabolic diseases and contributes substantially to disease initiation and progression. In obesity and T2DM, dysregulated macrophage activation promotes systemic inflammation and insulin resistance; in chronic obstructive pulmonary disease (COPD), defective phagocytic clearance by alveolar macrophages amplifies inflammatory burden; in MASLD, hepatic macrophage remodeling and inflammatory activation disrupts hepatic lipid metabolism and drives progression to metabolic dysfunction-associated steatohepatitis (MASH) and fibrosis; and in AS, foam cell formation and impaired macrophage migration accelerate plaque progression and instability (15–18). These findings underscore the central role of macrophage phenotypic plasticity in the pathogenesis of chronic metabolic diseases.

Emerging evidence indicates that essential transition metals are critical regulators of macrophage function, owing to their fundamental roles in cellular metabolism, signal transduction, and redox homeostasis. Maintenance of intracellular metal homeostasis is therefore indispensable for preserving macrophage functional integrity, whereas metal deficiency or overload can profoundly alter macrophage polarization, cytokine secretion, and key signaling pathways, including STAT3, nuclear factor kappa-B (NF-κB), and HIF-1α, ultimately leading to macrophage dysfunction. In addition, different forms of metal particles may further reshape the macrophage immune microenvironment by modulating reactive oxygen species (ROS) generation and cellular stress responses. Given these diverse regulatory effects, increasing attention has been directed toward understanding how essential transition metals influence macrophage phenotypes and functions, as well as how metal homeostasis imbalance contributes to the pathogenesis of chronic metabolic diseases through macrophage dysfunction. In the following sections, we comprehensively review the roles of major essential transition metals in macrophage biology and discuss the underlying mechanisms linking disrupted metal homeostasis to chronic metabolic disease progression.

### 2.2 The influence of iron on macrophages

Iron is indispensable for cellular metabolism, and its homeostasis is maintained through tightly coordinated processes governing iron uptake, storage, utilization, and export. Central to this regulation is the hepcidin–ferroportin (FPN) axis, in which hepcidin binds to FPN, the sole known cellular iron exporter expressed on enterocytes, macrophages, hepatocytes, and placental cells, and induces its internalization and degradation, thereby limiting iron efflux (19–21). Consequently, increased FPN expression promotes iron export and reduces intracellular iron storage, whereas hepcidin-mediated FPN degradation results in iron retention and intracellular iron accumulation. Under inflammatory conditions, activation of the BMP6–SMAD and STAT3 pathways enhances endogenous hepcidin production, leading to macrophage iron sequestration. Although this response is considered an adaptive component of innate immunity that restricts pathogen access to iron, increasing evidence suggests that iron retention also influences macrophage polarization and functional reprogramming. Thus, inflammatory cytokines such as IL-6 promote hepcidin expression, which degrades FPN and retains iron within macrophages; iron-loaded macrophages in turn release pro-inflammatory mediators that further stimulate hepcidin. This IL-6/STAT3–hepcidin–FPN–iron retention–macrophage activation axis constitutes a self-reinforcing feedback loop that links immune activation to disturbed iron homeostasis (22, 23). In addition to systemic regulation by the hepcidin–FPN axis, intracellular iron-handling molecules are also essential for preserving macrophage function. Among these, six-transmembrane epithelial antigen of the prostate 3 (STEAP3), a key metalloreductase involved in iron homeostasis, plays a critical role in maintaining proper intracellular iron distribution and the cytoplasmic labile iron pool. STEAP3 deficiency disrupts iron trafficking, impairs TLR4-mediated signaling, and reduces the production of interferon-β and chemokines, thereby compromising macrophage innate immune responses (24). Collectively, these findings indicate that both extracellular and intracellular iron regulatory mechanisms are indispensable for maintaining macrophage functional integrity, and that disruption of iron homeostasis may contribute to macrophage dysfunction in inflammatory and metabolic diseases.

On the basis of this regulatory framework, alterations in intracellular iron availability directly influence macrophage polarization and phenotypic plasticity. Macrophages are highly sensitive to fluctuations in iron levels, and different polarization states are associated with distinct iron-handling patterns (4, 25, 26). Macrophage polarization is closely associated with distinct iron-handling phenotypes. Classically activated M1-like macrophages generally exhibit reduced ferroportin (FPN) expression and increased ferritin-mediated iron sequestration, whereas alternatively activated M2-like macrophages show higher FPN expression and a greater capacity for iron export (25). However, the causal effects of intracellular iron availability on macrophage polarization are context dependent. In some experimental models, increased iron availability enhances the expression of M2-associated markers and attenuates LPS/TLR4-driven pro-inflammatory responses, whereas dietary iron deficiency exacerbates LPS-induced inflammatory cytokine production (25, 27, 28). Conversely, other studies have reported pro-inflammatory effects of iron loading, indicating that the relationship between iron status and macrophage polarization depends on the cellular, metabolic, and inflammatory context (29).Importantly, this relationship is further shaped by tissue-specific metabolic contexts. For example, iron-rich MFe^hi^ adipose tissue macrophages (ATMs) can act as a local iron buffer. Under chronic high-iron diet conditions, MFe^hi^ ATMs preferentially accumulate excess iron and upregulate the iron-storage genes *Fth1* and *Ftl1*, while retaining a predominantly M2-like, low-inflammatory phenotype. This specialized iron-handling capacity helps protect adipocytes from iron overload and contributes to local adipose tissue iron homeostasis (30).

Exogenous methods for metal intervention are currently the focus of research in immunopharmacology. deferiprone (DFP) is highly lipophilic and can penetrate cell membranes to enter cells; it can cross the blood-brain barrier and myocardial cell membranes to clear the intracellular labile iron pool (LIP), outperforming the hydrophilic DFO (deferoxamine) (31). By manipulating intracellular iron levels through pharmacological means, macrophage inflammatory responses can be modulated: iron chelators such as DFP have been shown to inhibit LPS-induced pro-inflammatory cascades, downregulate M1-associated markers such as iNOS, reduce nitric oxide and reactive oxygen species production, and significantly decrease the transcription and release of pro-inflammatory cytokines, including IL-1β, IL-6, and TNF-α. *In vivo*, treatment with DFP (80 mg/kg, intraperitoneally, once daily for 16 weeks) in mice reduced plaque burden. However, DFP treatment was also associated with mild anemia and reductions in serum iron and transferrin saturation, while plasma total cholesterol and triglyceride levels remained unchanged (32). Prussian blue nanoparticles (PBNPs) are commonly associated with an anti-inflammatory M2-like phenotype through IL-4/IL-13 alternative activation pathways (13, 33). However, these effects are highly context dependent and can be influenced by particle size, surface modification, chemical composition, and external stimuli; accordingly, certain engineered PBNPs have also been shown to reprogram tumor-associated macrophages (TAMs) toward an M1-like phenotype.

Importantly, the relationship between macrophage immune status and iron metabolism is bidirectional. On the one hand, iron-targeted interventions can reshape macrophage function; on the other hand, macrophage activation itself can feed back to regulate iron homeostasis. In the presence of low-density lipoprotein (ox-LDL), treatment of primary mouse peritoneal macrophages with 100 μM ferric ammonium citrate (FAC) for 48 h significantly increases intracellular iron accumulation and lipid deposition. FAC-induced iron overload is also associated with increased ROS production and enhanced oxidative stress, which may further affect the expression of liver X receptor α (LXRα) and its downstream cholesterol transporters ABCA1 and ABCG1, leading to impaired ApoAI-mediated cholesterol efflux and intracellular lipid retention (32) Meanwhile, FAC treatment can increase the secretion of pro-inflammatory cytokines (such as TNF-α and IL-1β) in macrophages and is associated with a more pronounced M1-like pro-inflammatory phenotype (34). In addition, iron accumulation and ox-LDL stimulation can promote the production and secretion of IL-6, and the secreted IL-6 may promote the transcription of hepcidin in hepatocytes by activating the JAK/STAT3 signaling pathway (22, 23). Conversely, Hereditary hemochromatosis is characterized by mutations in HFE, TfR2, or HJV that lead to insufficient hepcidin expression, disrupting the crosstalk between macrophage-derived cytokines and hepatocellular signaling, resulting in excessive intestinal iron absorption and hepatic iron deposition (23). Collectively, these findings identify macrophages as a central hub linking immune regulation and iron metabolism, and suggest that macrophage dysfunction driven by disturbed iron homeostasis may contribute to the pathogenesis of chronic metabolic diseases.

### 2.3 The influence of copper on macrophages

In the current body of literature, evidence for the physiological role of copper in macrophages remains relatively limited. Most mechanistic conclusions are derived from models of copper deficiency, copper overload, or inflammatory stimulation (LPS and IFN-γ), which do not fully reflect true steady-state physiological conditions. Based on the more consistent findings available, the core physiological function of copper in macrophages is likely to lie in maintaining intracellular metal homeostasis and supporting fundamental innate immune defense mechanisms.

First, copper is an essential enzymatic cofactor required for the basal biochemical functions of macrophages. For instance, it is critical for the activity of copper-dependent enzymes such as ceruloplasmin (Cp). Cp exhibits ferroxidase activity, catalyzing the oxidation of ferrous iron (Fe²^+^) to ferric iron (Fe³^+^), a necessary step for the safe export of iron from macrophages and its subsequent binding to transferrin (35). Beyond its role in ceruloplasmin-mediated iron metabolism, copper is also involved in regulating the expression of the iron export protein FPN. Experimental studies have shown that copper loading significantly enhances FPN transcription in primary bone marrow-derived macrophages (BMDMs) and can partially counteract LPS-induced suppression of FPN, suggesting a potential role in mitigating inflammation-associated anemia (36).

Pro-inflammatory signals remodel macrophage copper homeostasis. In RAW264.7 macrophages, IFN-γ induces CTR1 to boost copper uptake. ATP7A relocates from the Golgi to phagosomes, delivering copper into pathogen-containing vacuoles and raising phagosomal copper levels (35). Copper also exerts bidirectional regulatory effects on inflammatory responses. Excess copper exposure can promote inflammatory responses characterized by increased production of pro-inflammatory cytokines, including IL-6, TNF-α, and IL-1β. In parallel, intracellular copper is required for canonical NLRP3 inflammasome activation, which promotes caspase-1 activation and subsequent maturation and release of IL-1β. In contrast, at lower concentrations, copper has been reported to promote macrophage polarization toward the M2 phenotype, contributing to anti-inflammatory activity and tissue repair (37, 38). Although current evidence for the physiological role of copper in macrophages remains incomplete, available findings suggest that copper is essential for maintaining intracellular metal homeostasis, coordinating iron handling, and supporting context-dependent innate immune responses, thereby positioning copper as an important though still underexplored regulator of macrophage biology.

### 2.4 The influence of zinc on macrophages

Zinc serves as a key modulator of macrophage function, and its homeostasis is essential for preserving phagocytosis, intracellular pathogen clearance, and cytokine secretion. Accordingly, zinc deficiency disrupts these fundamental processes, leading to impaired host defense, dysregulating inflammatory responses, heightened susceptibility to infection, and aggravated tissue injury (39, 40). Mechanistically, zinc acts in part as an endogenous antioxidant, limiting superoxide anion generation through the suppression of NADPH oxidase activity and thereby protecting macrophages from oxidative stress induced damage. Recent studies further indicate that excessive ROS promote the formation of Macrophage extracellular traps (METs), DNA–protein web-like structures whose overproduction contributes to chronic inflammatory pathology. In this context, zinc suppresses ROS accumulation, inhibits PAD4 activation, and blocks the NLRP3/caspase-1/GSDMD pyroptotic axis, collectively restraining aberrant MET formation and mitigating inflammatory tissue damage (41). Moreover, zinc can directly inhibit the proteolytic activity of caspase-1, providing an additional layer of control over excessive inflammasome activation (42). Notably, this dose–response relationship is bidirectional. At nutritional oral doses (12–15 mg elemental zinc/day for 18 months), a randomized trial detected no adverse effects and no symptoms of zinc toxicity (43), whereas zinc excess disrupts mitochondrial redox balance, lysosomal function, and endoplasmic-reticulum homeostasis and can itself activate NLRP3 signaling; metallothioneins buffer surplus cytosolic zinc to prevent such cytotoxicity (44). Importantly, zinc intakes above the FDA Daily Value have conversely been associated with adverse immunological outcomes, indicating that benefits are confined to repletion of deficiency rather than indiscriminate supplementation (43).

Under physiological conditions, intracellular zinc homeostasis in macrophages is tightly regulated rather than freely diffusible. Its transmembrane transport primarily depends on the SLC39 (ZIPs) family mediating zinc influx, and the SLC30 (ZnTs) family mediating zinc efflux or sequestration into intracellular organelles. In the cytosol, metallothioneins serve as the main zinc-binding proteins and precisely regulate intracellular zinc concentrations by maintaining free zinc at picomolar levels (39, 45). For example, ZIP8 is constitutively present to preserve basal zinc homeostasis in quiescent macrophages; upon LPS or inflammatory stimulation, ZIP8 is strongly upregulated, increasing zinc influx to modulate NF-κB and MAPK signaling, thereby balancing pro-inflammatory cytokine (TNF-α, IL-6, IL-1β) production. ZIP8 cooperates with ZIP1 and ZIP2 to fine-tune intracellular zinc concentrations and macrophage responses, and its dysregulation impairs macrophage function, contributing to inflammatory disorders (16, 39, 46). In addition, lysosomes serve as important intracellular zinc storage compartments and can participate in zinc redistribution and release through mucolipin transient receptor potential channel 1 (MTC1) (47). Metallothioneins are not zinc selective: they also chelate copper, cadmium, mercury, and silver, providing a molecular basis for cross-metal competition (44). Several ZIP transporters are likewise shared metal conduits—ZIP14 transports both zinc and manganese, and its deletion causes cerebral manganese accumulation together with disturbed iron and glucose metabolism. The tissue-specific distribution of these transporters (ZnT8) in pancreatic insulin granules; ZIP14 in liver and intestine; ZIP8, ZIP13, and ZIP14 in adipose tissue) also defines where absorbed zinc is delivered and retained, providing a transporter-level basis for biodistribution (44).

### 2.5 The influence of cobalt on macrophages

In the physiological microenvironment of humans and other mammals, cobalt is an essential trace transition metal whose endogenous biological functions are mediated almost entirely through its role as the central atom of cobalamin (vitamin B12, Odaka). The role of endogenous cobalt in macrophages is primarily reflected in the participation of cobalt-containing vitamin B12 in macrophage immune responses and metabolic regulation. M1 macrophages preferentially generate energy through aerobic glycolysis and produce pro-inflammatory mediators, whereas M2 macrophages depend mainly on mitochondrial oxidative phosphorylation. As a cobalt-containing cofactor, vitamin B12 is required for the activity of mitochondrial methylmalonyl-CoA mutase (MUT), which catalyzes the conversion of L-methylmalonyl-CoA to succinyl-CoA and thereby supports normal tricarboxylic acid cycle function. Interestingly, a recent mechanistic model has linked macrophage vitamin B12 metabolism to the antimicrobial effects of itaconate. Pathogen-activated macrophages produce itaconate through ACOD1, and the resulting itaconyl-CoA can inhibit B12-dependent MUT and locally decrease vitamin B12 availability; this localized B12 depletion has been proposed to restrict the survival of macrophage-ingested bacteria (48). Although B12 deficiency may impair succinyl-CoA production and alter mitochondrial metabolism in macrophages, direct evidence linking B12 deficiency to reduced oxidative phosphorylation and subsequent M1 polarization remains limited. This potential metabolic link may contribute to inflammation and tissue injury, but requires further experimental validation (49). In LPS-stimulated BV2 microglial cells, vitamin B complex treatment reduced inflammatory responses associated with TLR4 signaling. In silico molecular docking further predicted a potential interaction between vitamin B12 and CD14, raising the possibility that vitamin B12 may interfere with CD14-mediated LPS presentation to the TLR4/MD2 complex. However, direct physical binding between vitamin B12 and CD14, as well as its functional blockade of LPS transfer, has not yet been experimentally demonstrated (50). Cobalt contributes to mammalian metabolism primarily as the central metal ion of cobalamin (vitamin B12), rather than as free cobalt. Vitamin B12 serves as an essential cofactor for methionine synthase (MTR), which remethylates homocysteine to methionine and thereby supports the generation of S-adenosylmethionine (SAM), a universal methyl donor for DNA, RNA, protein, and histone methylation. Vitamin B12 availability can therefore influence epigenetic regulation through one-carbon metabolism. Consistent with this mechanism, Kovatcheva et al. showed that vitamin B12 availability modulates SAM-dependent H3K36 trimethylation, transcriptional fidelity, cellular reprogramming, and tissue repair (51). Although one-carbon metabolism and SAM-dependent chromatin modifications are also important in macrophage activation and polarization, direct evidence that cobalt deficiency per se impairs macrophage-mediated tissue repair remains limited (51).

Exogenous or excessive cobalt exposure can cause marked cytotoxicity. This shift from a physiological cofactor to a pathological agent disrupts macrophage function through several mechanisms. First, cobalt induces inflammatory activation *in vitro*: high concentrations of cobalt nanoparticles (CoNPs) significantly increase the secretion of TNF-α and IL-1β, and HIF-1α appears to participate in cobalt-induced cytotoxic responses (52). Second, cobalt impairs macrophage migration *in vitro*. Both Co²^+^ and CoNPs promote macrophage spreading, podosome formation, and extracellular matrix adhesion by downregulating RhoA signaling through ROS-dependent mechanisms, thereby retaining macrophages at inflammatory sites and delaying the resolution of inflammation (53). Third, cobalt activates hypoxia-related signaling. Cobalt ions mimic hypoxia by stabilizing HIF-1α and thereby activate hypoxic signaling under normoxic conditions. However, HIF-1α silencing increases TNF-α and IL-1β production without alleviating cytotoxicity, suggesting that cobalt-induced macrophage apoptosis occurs through an HIF-1α-independent mechanism (52). Notably, the direct pathogenic factors of HIF-1α-independent apoptosis remain uncharacterized, representing an important research gap.

### 2.6 The influence of manganese on macrophages

For a long time, academic research on manganese has been highly confined to the neurotoxicity caused by exogenous exposure (such as manganese poisoning), while studies on how manganese, at physiological concentrations, functions as an endogenous signaling molecule to participate in the regulation of oxidative stress and inflammatory responses in macrophages are almost non-existent. In macrophages, manganese ions not only act as cofactors for antioxidant enzymes but also serve as natural immune activators, directly participating in the regulation of the cGAS–STING signaling pathway and the ROS–apoptosis network. However, current mechanistic studies on the effects of manganese on macrophages rely largely on *in vitro* cell lines and probe-based techniques, which have certain limitations in faithfully recapitulating the complex *in vivo* homeostasis of metal ions and the immune microenvironment (54).

To summarize the mechanisms by which transition metals regulate macrophage function, **Table 1** compiles representative molecular targets and signaling pathways through which different transition metals influence macrophage biology, together with the corresponding authors, thereby providing an integrated framework for further understanding their roles in the development and progression of chronic inflammatory and metabolic diseases.

**Table 1:** Molecular targets and signaling pathways mediating the effects of transition metals on macrophage function.

Transition metal	Molecular target/pathway	Authors	Evidence source of the pathway
Fe	Hepcidin-Ferroportin axis	19; 20; 21	Animal + primary-cell + cell-line (Babitt)
	Bmp6-Smad and STAT3 pathways	19; 20; 21; 24; 22	Cell-line (Zhang)human + animal (Li)
	Fth1, Ftl1 (Iron storage genes)	30; 55	Animal + primary-cell (Hubler)
	Arg1, iNOS, and Ferroptosis	14	Animal
	IRF5 pathway	13 33	Animal + cell-line (Gu)
	HFE, TfR2, HJV	23	Cell-line
Cu	Ceruloplasmin	35	Cell-line
	Fpn	36	Primary-cell
	CTR1 and ATP7A	35	Cell-line
	NF-κB, MAPKs, NLRP3 inflammasome	37, 38	Animal
	IL-1β, IL-6, TNF-α	38; 37	Animal (Liu)
Zn	NLRP3/caspase-1/GSDMD pyroptosis pathway	41 47	Animal+ cell-line+ nanoparticle (Hu)
	Caspase-1	42	animal+ primary-cell+ cell-line
	SLC39 (ZIPs), SLC30 (ZnTs), Metallothioneins	39 45	Animal
	ZIP8, ZIP1, ZIP2	16	Human + animal + primary-cell
	NF-κB and MAPK signaling	16	Human + animal+ primary-cell
	MTC1	47	Nanoparticle
	TNF-α, IL-6, IL-1β	46;	Human + primary-cell (Pyle)
		56;	
		57	
	A20/IKK-α/NF-κB pathway	41;	Primary-cell + cell-line (Prasad)
		56;	
		57;	
		58	
	ZnT8	59	Human
	MUT/TCA cycle	49	Human Cell-line Animal + primary-cell
	MS/MTR/SAM	51	
Co	CD14 receptor/TLR4 pro-inflammatory pathway	50	Cell-line
	HIF-1α pathway	52	Cell-line + nanoparticle
	RhoA signaling pathway/ROS	53	Animal + primary-cell + cell-line+ nanoparticle
	cGAS–STING signaling pathway	54	Human + primary-cell (Mokgobu)
	RhoA signaling pathway/ROS	1	Animal + primary-cell + cell-line+ nanoparticle
Mn	cGAS–STING signaling pathway ROS–apoptosis network	54	Human + primary-cell (Mokgobu) Human+ primary-cell

### 2.7 Summary

Despite their distinct biochemical properties and metal-specific regulatory systems, essential transition metals influence macrophage biology through several partially overlapping processes. A recurring feature is altered metal availability, trafficking, or intracellular compartmentalization. Iron homeostasis is regulated principally through the hepcidin–FPN axis together with intracellular iron-handling proteins such as STEAP3 (19, 20, 23, 24). Zinc homeostasis depends largely on the SLC39 (ZIP) and SLC30 (ZnT) transporter families and metallothioneins (39, 60), whereas copper distribution is regulated by transporters including CTR1 and ATP7A and is remodeled during macrophage activation (35, 36). Cobalt differs from these metals because its physiological functions in mammals are mediated predominantly through cobalamin-dependent biochemistry (61); manganese serves as a cofactor for enzymes including manganese superoxide dismutase and has also been implicated in innate immune signaling (62, 63). Thus, although the molecular machinery differs substantially among metals, changes in their availability or intracellular distribution can modify metal-dependent metabolic and immune functions. The consequences of such changes, however, are metal- and context-specific rather than uniform.

These homeostatic systems are also interconnected. Copper-dependent ceruloplasmin possesses ferroxidase activity and facilitates the oxidation of Fe²^+^ to Fe³^+^, thereby supporting iron efflux from macrophages and subsequent loading onto transferrin (64). Metallothioneins can bind multiple metal species, including zinc and copper (65); some metal transporters exhibit overlapping substrate specificity; ZIP14, for example, can transport zinc, manganese, and non-transferrin-bound iron (66). These interactions provide a molecular basis through which altered handling of one metal may influence the intracellular availability or biological activity of another (65, 66). Accordingly, macrophage metal regulation is better viewed as an interconnected network of metal-handling pathways rather than fully independent systems. Nevertheless, the magnitude and physiological relevance of these cross-metal interactions likely depend on the transporter, tissue and biological context.

Changes in intracellular metal availability can modify ROS generation, antioxidant capacity, mitochondrial function, although the direction and biochemical basis of these effects differ among metals. In macrophages exposed to pro-oxidative conditions such as oxidized ox-LDL, iron accumulation is associated with increased ROS production, enhanced lipid deposition, and impaired cholesterol efflux through alterations involving LXRα and its downstream transporters ABCA1 and ABCG1 (32). Physiological zinc contributes to antioxidant defense, in part by limiting NADPH oxidase-dependent superoxide generation, whereas excessive zinc can itself disturb cellular and redox homeostasis (39, 44). Exogenous Co²^+^ and cobalt-containing particles can also induce ROS-dependent responses; in particular, ROS-mediated suppression of RhoA signaling has been associated with increased macrophage spreading, adhesion, and retention at inflammatory sites (53). Manganese further illustrates the context dependence of metal–redox interactions: manganese is required for the catalytic activity of mitochondrial manganese superoxide dismutase (MnSOD) (62), whereas excessive manganese exposure has been associated with increased ROS or H_2_O_2_ production and cellular stress (54). Copper excess, meanwhile, has been associated with inflammatory activation, although the macrophage mechanisms identified to date are not necessarily identical to the ROS-centered pathways described for iron, zinc, or cobalt (37, 38). Redox disturbance should therefore be considered a recurrent, but neither universal nor mechanistically identical, link between altered metal handling and macrophage dysfunction.

Downstream of these metabolic and redox changes, several innate immune signaling pathways recur across different metal systems. NF-κB- and MAPK-related signaling is particularly prominent in studies of zinc and copper, whereas changes in iron handling can modulate TLR4-associated responses and inflammatory cytokine production (24, 37, 38, 46). For example, ZIP8-mediated zinc redistribution during inflammatory stimulation can modify NF-κB and MAPK signaling and thereby influence cytokine secretion (46). Intracellular copper availability has also been directly linked to canonical NLRP3 inflammasome activation and subsequent IL-1β maturation (67). In iron-loaded macrophages, increased oxidative stress can coincide with enhanced production of inflammatory mediators including TNF-α, IL-1β, and IL-6 (22, 32). Other metals engage more distinct signaling pathways. Manganese can enhance cGAS–STING signaling by increasing the sensitivity of cGAS to cytosolic DNA and promoting downstream STING activation (63), although direct evidence defining the physiological importance of this mechanism specifically in macrophages remains comparatively limited. In contrast, cobalt exposure can engage HIF-1α-associated responses as well as ROS/RhoA-dependent changes in macrophage adhesion and migration (52, 53). Thus, rather than acting through a single universal downstream pathway, different metals appear to intersect with a limited set of inflammatory and stress-responsive signaling modules whose relative contributions vary according to metal identity and experimental context. These signaling and metabolic changes are ultimately reflected in macrophage functional reprogramming, including alterations in cytokine secretion, phagocytosis, antimicrobial activity, migration, cellular metabolism, and polarization-associated phenotypes. The direction of macrophage reprogramming is highly context dependent and can vary with metal identity, concentration, chemical form, duration of exposure, subcellular localization, tissue microenvironment, and the pre-existing activation state of the macrophage. This context dependence is particularly evident for iron, zinc, and copper, for which physiological availability supports normal cellular functions, whereas deficiency, overload, or abnormal redistribution can produce distinct and, in some experimental settings, apparently opposing macrophage phenotypes (30, 37–39). Consequently, observations obtained under one exposure condition should not be generalized to all concentrations, chemical forms, or biological contexts of a given metal.

Finally, the relationship between metal metabolism and macrophage activation is bidirectional. Inflammatory stimuli can alter the expression or localization of metal-handling proteins, thereby changing intracellular metal availability, while the resulting changes in metal distribution can in turn modify metabolic, redox, and inflammatory responses. For example, IL-6/STAT3 signaling increases hepcidin expression, which subsequently promotes FPN internalization and degradation and favors cellular iron retention (22, 23). IFN-γ stimulation increases CTR1 expression and promotes ATP7A redistribution from the Golgi toward phagosomal compartments, thereby remodeling cellular and phagosomal copper handling (35). Inflammatory stimulation likewise induces ZIP8-dependent changes in zinc influx and intracellular zinc availability, which can feed back on inflammatory signaling (46). These observations support reciprocal coupling between inflammatory activation and metal homeostasis. However, the strength and direction of this coupling differ among metals, and positive-feedback relationships have been proposed or demonstrated only in specific settings, particularly in the IL-6/STAT3–hepcidin–FPN–iron-retention axis, rather than being established as a universal property of all essential transition metals (22, 23). Taken together, altered metal handling, redox and metabolic perturbation, innate immune signaling, and macrophage functional reprogramming provide an integrative framework for understanding how essential transition metals may contribute to chronic inflammatory and metabolic diseases.

## 3 Metal-induced macrophage dysfunction in chronic diseases

### 3.1 Metal-induced immune dysregulation in diabetes mellitus

Diabetes mellitus encompasses a spectrum of metabolic disorders defined by chronic hyperglycemia. It is broadly categorized into type 1 diabetes mellitus (T1DM) and T2DM. T1DM represents a persistent autoimmune condition characterized by immune-mediated destruction of pancreatic β-cells. When most pancreatic β-cells have been destroyed, the pancreas becomes unable to produce sufficient insulin in response to circulating glucose levels (9, 56). T2DM is characterized by impaired insulin secretion and glucose intolerance, often accompanied by compensatory hyperinsulinemia in the early stages, and its pathogenesis is closely associated with innate and adaptive immune factors that drive insulin resistance (18). The dysfunction of macrophages caused by the imbalance of the stable state of transition metals plays a particularly significant role in regulating inflammatory responses, β-cell function, and insulin sensitivity, especially in the case of T2DM.

In T2DM, obesity is a key risk factor, and adipose tissue inflammation driven by macrophage dysfunction is a core pathological process. In obesity and T2DM, adipose tissue macrophages shift toward the M1-like inflammatory state described above, increasing TNF-α, IL-1β, and IL-6 and activating JNK/NF-κB–iNOS signaling, thereby aggravating systemic insulin resistance (56). In contrast, M2 macrophages counteracts the pathological actions of TNF-α in adipose tissue. They also exhibit elevated expression of Arg1, an enzyme that competes with iNOS for the substrate L-arginine, thereby reducing nitric oxide production and attenuating inflammatory responses (56). Taken together, these findings suggest that, beyond classical metabolic and immune mechanisms, disturbances in the homeostasis of key transition metal elements may represent an additional layer of regulation in T2DM, potentially influencing macrophage function, chronic inflammation, and systemic insulin resistance.

In clinical settings, patients with T2DM typically exhibit abnormally elevated serum ferritin levels (68). Under physiological conditions, the liver secretes hepcidin to limit iron entry into the circulation. However, in T2DM, this classical feedback mechanism is disrupted. Studies have shown that although serum iron and ferritin levels are significantly increased in both T2DM patients and diabetic mouse models, serum hepcidin levels are paradoxically decreased (69). In the livers of db/db mice, although the BMP/SMAD signaling pathway successfully senses elevated circulating iron and becomes activated, it fails to effectively induce hepcidin transcription. This phenomenon, in which metabolic disturbances override normal iron-sensing signals, is referred to as iron resistance (69). Under physiological conditions, iron participates in mitochondrial ATP production and moderate ROS generation, both of which are essential for maintaining glucose-stimulated insulin secretion (GSIS). However, when iron homeostasis is disrupted, excess iron induces excessive oxidative stress via the Fenton reaction and promotes abnormal degradation of HIF-1α, thereby impairing β-cell function and suppressing insulin secretion (70). Approximately 25% of resident macrophages exhibit significantly elevated intracellular iron levels (approximately twice that of other macrophages). These cells are referred to as MFe^hi^ macrophages and are characterized by high expression of heme oxygenase-1 (HO-1). Under conditions of iron overload, MFe^hi^ macrophages can sequester excess iron, thereby protecting adipocytes from iron-induced toxicity, reducing oxidative stress and pro-inflammatory cytokine production, and indirectly improving insulin signaling (34). However, in T2DM, the function of these cells collapses, leading to the leakage of free iron into adipocytes, which induces lipid peroxidation and reduces adiponectin levels. This, in turn, exacerbates adipose tissue inflammation and promotes insulin resistance (34, 71). These alterations establish a feedback loop in which hyperglycemia and inflammation impair hepcidin regulation, leading to macrophage iron retention; iron-loaded macrophages then exacerbate adipose inflammation and insulin resistance, which further disturbs iron homeostasis. The future therapeutic direction could be to rescue this group of cells through microenvironment immune remodeling. Although the prevailing view suggests that iron overload aggravates T2DM, some animal studies indicate that its effects are highly cell type–specific. Paeschke et al. reported that in db/db mice, a model of severe metabolic dysregulation, a high-iron diet significantly increased pancreatic β-cell area, insulin immunofluorescence intensity, and serum insulin levels, thereby effectively reducing blood glucose and HbA1c. These findings suggest that supplementation with non-heme iron may support β-cell survival and insulin biosynthesis by correcting intracellular micronutrient imbalances. Furthermore, during the progression of diabetic peripheral neuropathy, a high-iron diet significantly reduced CD11c^+^ pro-inflammatory M1 macrophages in peripheral nerves while increasing CD163^+^ anti-inflammatory M2 macrophages. Previous studies have shown that appropriate levels of iron can inhibit M1 macrophage polarization and reduce the production of pro-inflammatory cytokines such as TNF-α, thereby alleviating local inflammatory responses in the neural microenvironment and improving nerve conduction function (55). To delineate the mechanistic link between iron homeostasis imbalance, macrophage dysfunction, and insulin resistance in obesity and T2DM, a dual-axis schematic is presented in **Figure 2**.

**Figure 2:**
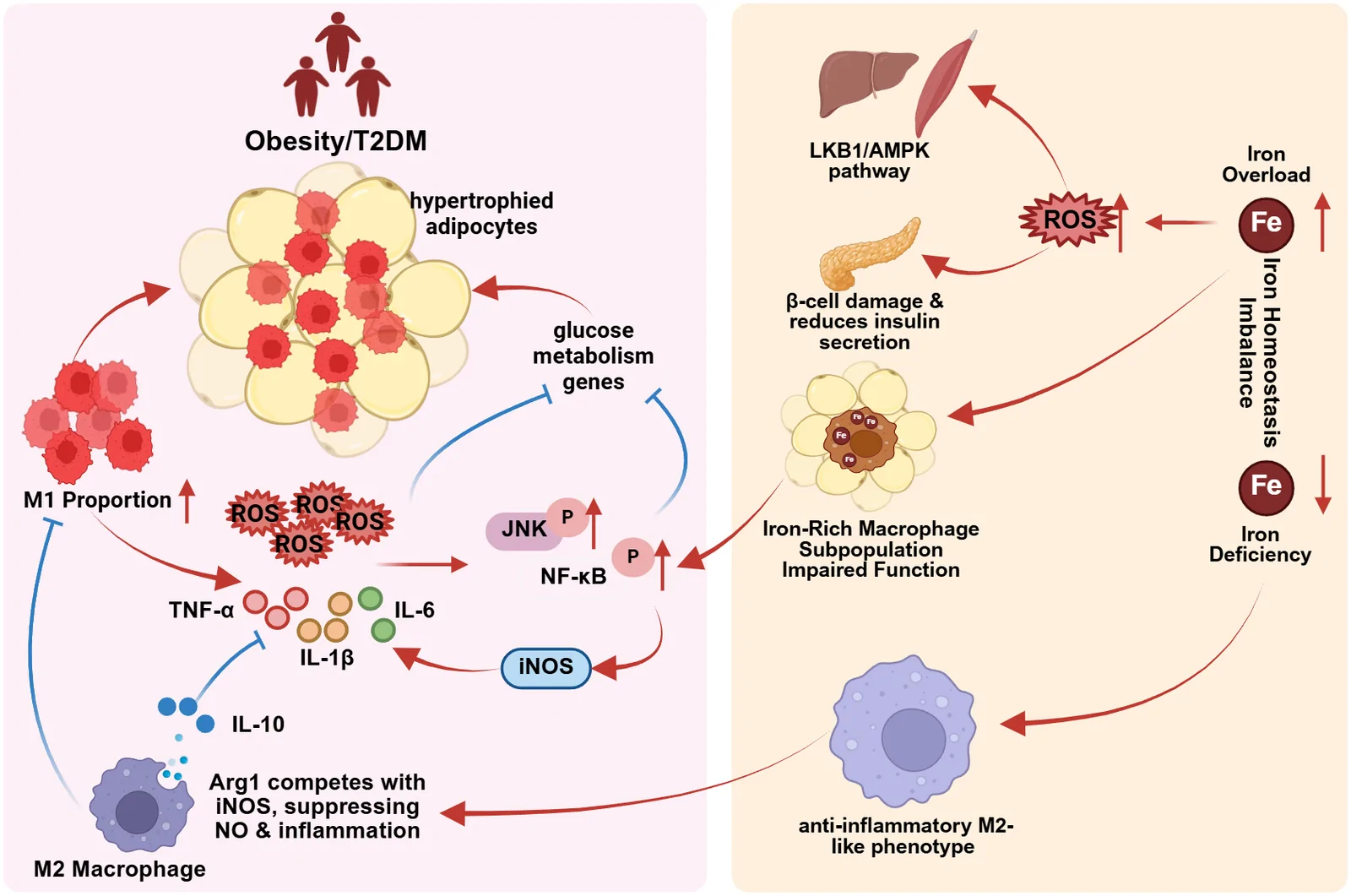
Iron homeostasis imbalance modulates macrophage polarization and drives adipose tissue inflammation in obesity and type 2 diabetes mellitus. Schematic was created with [BioRender.com] (https://BioRender.com).

During the onset and progression of T2DM, a significant disruption of zinc homeostasis occurs, representing an important endogenous driver of metabolic dysfunction. First, diabetes-associated gastrointestinal alterations impair intestinal zinc absorption. Second, hyperglycemia and metabolic disturbances interfere with the active reabsorption of zinc by renal tubular cells, leading to hyperzincuria (72) Clinical studies consistently demonstrate that serum zinc levels in patients with T2DM are significantly lower than those in healthy individuals, whereas urinary zinc excretion is pathologically elevated. This “hyperzincuria” is thought to result from impaired renal tubular zinc reabsorption combined with osmotic diuresis caused by hyperglycemia (73). In healthy pancreatic β-cells, ZnT8 (SLC30A8) is responsible for transporting cytosolic zinc ions into insulin secretory granules, where two zinc ions coordinate with six insulin molecules to form stable zinc–insulin hexamer crystals, a process essential for insulin maturation and storage (59, 72). In pancreatic β-cells, ZnT8, encoded by SLC30A8, transports zinc into insulin secretory granules, where zinc is required for insulin crystallization and efficient hormone packaging. Studies using ZnT8-knockout mice demonstrated a marked reduction in mature dense-core insulin granules and impaired insulin crystallization. Although glucose homeostasis and glucose-stimulated insulin secretion were largely preserved under basal dietary conditions, ZnT8-deficient mice developed impaired glucose tolerance and reduced islet responsiveness when challenged with a high-fat diet, indicating that ZnT8 becomes particularly important under metabolic stress (74). Variants in SLC30A8 are also associated with altered susceptibility to T2DM, although their functional consequences are complex and cannot be explained simply by reduced ZnT8 activity. Beyond its role in β-cell insulin storage, zinc can exert insulin-mimetic and insulin-sensitizing effects in peripheral tissues. Zinc enhances insulin signaling in part by inhibiting negative regulators such as protein tyrosine phosphatase 1B (PTP1B) and PTEN, thereby facilitating PI3K/Akt activation, GLUT4 translocation, and cellular glucose uptake. Accordingly, inadequate zinc availability may weaken these signaling mechanisms and contribute to impaired peripheral glucose utilization and insulin resistance in T2DM (72, 74).

At this stage, reduced intracellular zinc levels have been associated with macrophage polarization toward a pro-inflammatory M1 phenotype (1, 75). These macrophage-derived pro-inflammatory factors can enter the systemic circulation via paracrine signaling and reduce the expression of GLUT4 in peripheral target cells, thereby markedly exacerbating systemic insulin resistance (56). Studies have shown that in high-fat diet–induced obesity models, concomitant zinc deficiency amplifies pathological changes in adipose tissue, leading to significantly increased expression of macrophage-specific markers (ADAM8, CD68, and F4/80), indicating markedly enhanced macrophage infiltration (76). Physiological concentrations of zinc can induce the expression of the anti-inflammatory protein A20 and directly or indirectly inhibit the activity of IKK, thereby preventing NF-κB translocation into the nucleus and subsequent inflammatory cascade activation (58, 72, 77). However, under zinc-deficient conditions in T2DM, macrophages lose their ability to suppress NF-κB and TLR4 downstream signaling pathways, resulting in uncontrolled cytokine release and oxidative burst. Therefore, exogenous zinc supplementation may modulate the signaling networks of immune cells such as macrophages, with ‘insulin-mimetic’ effects supported mainly by cell-based and preclinical studies. Mechanistically, appropriate zinc supplementation inhibits the activity of protein tyrosine PTP1B and PTEN, thereby activating the PI3K/Akt pathway, promoting GLUT4 membrane translocation, and increasing glucose uptake (78). In clinical applications, the use of a moderate dose of zinc sulfate (ZnSO_4_) regimens have been reported to modestly improve glycemic control in patients with type 2 diabetes mellitus; for example, doses of 22 mg/day for 16 weeks or 30 mg/day for 12 weeks were associated with reductions in HbA1c and improvements in the HDL ratio. In addition to glycemic control, these dosages also improved lipid profiles by reducing total cholesterol and optimizing the HDL ratio (78). Nevertheless, these effects are modest and somewhat inconsistent across trials, and meta-analyses of randomized controlled trials indicate that the overall quality of the evidence is low to moderate, with considerable heterogeneity in dose, formulation, and outcome measures (79, 80). Moreover, whether any glycemic benefit of zinc is mediated by restoration of macrophage function in humans has not been established. This may all be closely related to the process of macrophage function repair and regulation of the immune response. In the field of drug development, researchers have synthesized novel organic zinc complexes such as zinc bis(maltolato) [Zn(Mal)_2_], which, due to their specific coordination structure, exhibit stronger insulin-mimetic effects than inorganic zinc. Early studies demonstrated that Zn(Mal)_2_ inhibits epinephrine-stimulated release of free fatty acids (FFAs) from rat adipocytes, with a half-maximal inhibitory concentration (IC_50_;) of approximately 0.59 mM (81). Subsequent animal studies further showed that Zn(Mal)_2_ lowers blood glucose levels and improves glucose tolerance in KK-Ay mice with type 2 diabetes (82). Notably, the inhibitory effect of Zn(Mal)_2_ on adipocyte FFA release may also influence the immune microenvironment of adipose tissue. Under conditions of obesity and insulin resistance, excessive adipocyte lipolysis increases local and circulating FFA levels, and long-chain saturated fatty acids can enhance pro-inflammatory activation of macrophages. Adipocyte–macrophage co-culture studies have shown that adipocyte-derived saturated FFAs promote TLR4/NF-κB-associated inflammatory signaling and increase the production of inflammatory mediators such as TNF-α (83). Therefore, by reducing adipocyte-derived FFA release, Zn(Mal)_2_ may lower local saturated fatty acid exposure and consequently attenuate macrophage inflammatory activation, thereby limiting the production of pro-inflammatory cytokines and potentially reducing macrophage accumulation in adipose tissue. However, long-chain saturated fatty acids are now considered unlikely to act as direct TLR4 ligands; rather, TLR4-dependent metabolic and membrane lipid reprogramming appears to facilitate lipid-induced macrophage inflammation (84). Direct evidence that Zn(Mal)_2_ suppresses pro-inflammatory macrophage polarization or macrophage infiltration into adipose tissue is still lacking, and these immunomodulatory effects should therefore be regarded as a plausible but unconfirmed mechanism.

Despite advances in understanding the role of metal-induced macrophage dysfunction in diabetes, several knowledge gaps remain. First, the temporal dynamics, reversibility, minimal threshold, and optimal concentration of iron for maintaining specific macrophage phenotypes in diabetes are unclear. Second, the precise roles of different macrophage subpopulations and their interconversion mechanisms during diabetes progression, particularly the molecular basis for MFe^hi^ dysfunction in obesity, are not fully elucidated. Notably, although HO-1 has traditionally been regarded as an antioxidant protective factor, studies have shown that macrophage-specific deletion of HO-1 in a high-fat microenvironment can paradoxically reduce inflammation and improve insulin resistance, suggesting its strongly context-dependent role (85). Third, the integrative mechanisms by which the nervous, endocrine, and immune systems coordinate iron-metabolism signals in diabetes complications remain poorly understood. For example, it is still unclear how endocrine hormones such as hepcidin (secreted by the liver) and adiponectin (secreted by adipocytes) remotely regulate iron uptake and release in macrophages located within distal neural tissues via the circulation. Fourth, although basic research suggests that zinc may participate in the pathogenesis of T2DM through modulation of macrophage function, there is still a lack of large-scale, long-term prospective clinical evidence to support this hypothesis. Therefore, further studies are urgently needed to determine whether zinc supplementation or targeting zinc transporters (such as ZIP8) can produce definitive macrophage-mediated protective effects in humans and to evaluate their potential for clinical translation.

### 3.2 Metal-induced immune dysregulation in MASLD

The liver functions not only as the body’s largest metabolic organ but also as a key immune organ. Globally, MASLD, originally known as Non-alcoholic fatty liver disease, affects roughly 25–33% of adults in industrialized nations, with a global prevalence of about 25.2%. In China, the prevalence of MASLD ranges from 6.3% to 27%, accounting for 30% of global MASLD cases (8, 86). In Western countries, MASLD has become the most common liver disease. The typical pathogenesis of chronic liver disease generally progresses from MASLD to MASH, with approximately 20% of MASLD patients eventually developing cirrhosis (87). Hepatic macrophages comprise tissue-resident Kupffer cells (KCs) and monocyte-derived macrophages (MoMFs), which play distinct roles in MASLD. Resident lymphocytes and Kupffer cells (KCs) account for 10–20% of the total liver volume, and KCs are the primary cells mediating nonspecific phagocytosis in the liver, representing 80% of tissue-resident macrophages in the human body (88, 89). Embryonically derived KCs reside in liver sinusoids and are maintained mainly through CSF-1/CSF-1R-dependent local self-renewal under homeostatic conditions, contributing to pathogen clearance, iron recycling and lipid homeostasis (90). Research has shown that KCs are closely involved in the pathogenesis of MASLD/MASH, playing key roles from the steatosis stage to the inflammation stage and finally to the fibrosis stage (8). KCs secrete the anti-inflammatory cytokine IL-10 to maintain an anti-inflammatory microenvironment, but they can also excessively release inflammatory mediators including TNF-α, IL-1, IL-6, macrophage inflammatory protein-1α (MIP-1α), TGF-β, and RANTES, causing parenchymal cell injury and necrosis and even activating inflammatory cascades in neighboring cells (87, 89, 91). During MASLD progression, hepatocyte injury-associated danger signals and chemokine pathways such as CCL2/CCR2 promote circulating monocyte recruitment into the liver, where local microenvironmental cues drive their differentiation into distinct functional states of MoMFs (92). Inflammatory MoMFs produce mediators such as TNF-α, IL-1β and IL-6, exacerbating hepatic inflammation and metabolic dysregulation. In response to lipid accumulation and dying hepatocytes, a subset of MoMFs acquires lipid-associated macrophage (LAM) states characterized by the expression of markers such as triggering receptor expressed on myeloid cells 2 (TREM2) (93). These cells participate in lipid handling and tissue injury responses, highlighting the functional heterogeneity of hepatic macrophages during disease progression. Studies have shown that obesity and lipid accumulation are associated with M1-predominant polarization of KCs (94). In C57BL/6 mice fed a 60%-fat high-fat diet for 16 weeks, hepatic steatosis was accompanied by a preferential increase in CD11c^+^ M1-like KCs, together with increased expression of iNOS2, TNF-α, and IL-6 and enhanced secretion of TNF-α, IL-6, and IL-1β. Treatment with the PPAR-γ agonist rosiglitazone (30 mg/kg/day by oral gavage for 28 days) attenuated M1-associated KC activation and local inflammation (95). However, another study found that during NASH progression, hepatic M1 markers, including iNOS, IL-12p40, and CXCL10, peaked early and then declined, whereas M2 markers arginase-1 and MGL-1 remained relatively unchanged. Meanwhile, enlarged lipid-laden macrophages with increased IL-10 and annexin A1 emerged, and more importantly, similar annexin A1^+^ vacuolated macrophages were observed in human MASH biopsies (96). In the original single-cell study by Xiong et al. (97), scRNA-seq was performed on hepatic non-parenchymal cells from AMLN diet-induced MASH mice, yielding a total of 33,168 single-cell transcriptomes. The authors further characterized Kupffer cells and monocyte-derived macrophages. This study identified TREM2^+^GPNMB^+^ CD9^+^ MASH-associated macrophages (NAMs). Over 93% of TREM2^+^ Kupffer cells were derived from MASH livers, and flow cytometry validation demonstrated that GPNMB^+^CD9^+^ cells accounted for more than 60% of Kupffer cells in MASH. In a human MASH cohort of 144 subjects, hepatic TREM2^+^ expression was significantly correlated with inflammation, steatosis, hepatocyte ballooning, fibrosis, and NAS (97). This population exhibits composite transcriptional signatures linked to phagocytosis, lysosomal degradation, and antigen presentation. Accordingly, the classical M1/M2 binary framework is insufficient for investigating macrophages in MASLD. Future research should focus on the heterogeneity and specific functions of disease-associated macrophage subsets. Although recent studies have begun to dissect the roles of TREM2^+^/GPNMB^+^ macrophages in MASLD/MASH, mechanistic and human studies exploring the origin, function, and association with inflammatory severity of these novel macrophage subsets across different MASLD stages remain relatively scarce when compared with investigations on canonical M1/M2 polarization.

In MASLD, dysregulation of iron metabolism is one of the key endogenous pathological features. Clinically, this is often characterized by elevated serum ferritin levels, while transferrin saturation remains relatively normal, a phenomenon referred to as “metabolic dysregulated iron overload syndrome” (DIOS) (98). At the tissue level, hepcidin suppresses the export of iron by binding to and promoting the degradation of FPN on cell membranes. In the context of MASLD, chronic inflammation in the MASLD microenvironment often results in abnormal regulation of hepcidin expression. On one hand, obesity-related chronic low-grade inflammation and leptin secreted from adipose tissue can significantly upregulate hepcidin expression in hepatocytes through JAK2/STAT3 signaling pathways. Increased hepcidin levels accelerate the degradation of FPN on the surface of hepatocytes and KCs, causing iron to be “retained” in the liver, leading to local iron overload (98). On the other hand, in some MASH animal models (such as high-fat/high-cholesterol or high-fructose/low-copper diets), a reduction in hepcidin expression has been observed. This hepcidin deficiency lifts the inhibition on intestinal iron absorption, resulting in excessive absorption of dietary iron, thereby triggering systemic iron overload (99).

At the subcellular level, iron overload induces intracellular calcium ion accumulation, activating calcineurin, which dephosphorylates and translocates the autophagy-regulating factor TFEB into the nucleus, enhancing ferritin autophagy. Under conditions of iron overload, this mechanism, which is originally used to regulate iron storage, accelerates ferritin degradation, releasing large amounts of free iron into unstable iron pools, further amplifying oxidative stress responses, inducing lipid peroxidation, and may contribute ferroptosis (100).

Liver macrophage populations are highly sensitive to both iron and lipid toxicity, and endogenous iron metabolic dysregulation can drive their remodeling. Under physiological conditions, embryo-derived resident Kupffer cells (EmKCs) efficiently recycle iron from senescent red blood cells and maintain homeostasis under the regulation of transcription factors such as SPI-C and NRF2 (101). However, in the MASLD microenvironment, when free iron overload combines with lipotoxicity, this buffering system is rapidly overwhelmed. Importantly, iron accumulation itself can directly reshape KC phenotypes. In MASH mice, Kanamori et al. identified an iron-rich KC population (Fe-hi KCs) characterized by enhanced pro-inflammatory and pro-fibrotic programs. Mechanistically, iron accumulation activated MiT/TFE transcription factors, whereas iron chelation attenuated liver fibrosis (102). These findings indicate that local iron accumulation can actively contribute to inflammatory and fibrogenic reprogramming of hepatic macrophages. Iron-dependent lipid peroxidation significantly increases, potentially contributing, together with other metabolic stresses, to dysfunction and loss of resident KCs. The loss of resident KCs not only weakens the liver’s anti-inflammatory barrier but also amplifies local inflammatory responses through the release of damage-associated signals. As EmKCs decrease, vacancies emerge within the hepatic macrophage niche, and monocytes from the bone marrow are persistently recruited into the liver under chemokine signals. These recruited monocytes first differentiate into MoMFs, and under sustained local lipid, cytokine, and other niche signals, a subset undergoes transcriptional and epigenetic reprogramming and progressively acquires KC-like features, giving rise to monocyte-derived Kupffer cells (MoKCs). Although iron loading can markedly remodel KC phenotype, direct evidence that iron deposition itself drives the MoMF-to-MoKC transition remains lacking. However, other recruited MoMF populations may retain strong pro-inflammatory and pro-fibrotic programs, which, while involved in clearing metabolic products, also exacerbate the progression of MASH (94, 103). Collectively, iron overload, lipid peroxidation, Kupffer cell ferroptosis, and monocyte-derived macrophage recruitment form a feed-forward loop that perpetuates hepatic inflammation and fibrosis.

It is worth noting that traditionally, KCs have been viewed as significant drivers of liver injury, but animal experiments have provided conflicting evidence. Song et al. demonstrated that after depletion of KCs using gadolinium chloride (GdCl_3_) in a model mouse, although hepatic steatosis was significantly suppressed, overall liver injury and iron overload were not improved (99). The authors attributed this to a low copper state induced by CuMF (copper-deficient) diet, which disrupted the iron efflux process, leading to abnormal iron deposition primarily in hepatocytes, accompanied by systemic iron metabolic dysregulation caused by reduced hepcidin. Therefore, even in the absence of KCs, the low-copper microenvironment and the iron load within hepatocytes could not be reversed or cleared. This strongly suggests that, in future therapies aimed at improving iron overload in MASLD, failing to address the homeostasis of metals like iron and copper within the liver may undermine the overall therapeutic efficacy.

*In vitro* or isolated iron-modulation experiments indicate that the effect of iron on macrophage polarization depends on the microenvironment. In the chronic inflammatory and lipid-loaded environment of MASLD, pro-inflammatory cytokines such as IL-6 induce hepatocyte hepcidin production, leading to iron retention within KCs. Under this pathological context, iron overload promotes M1 polarization and inflammatory cytokine release, forming a vicious cycle that accelerates the progression of MASLD to MASH (104). Accordingly, iron overload, together with lipid accumulation, oxidative stress, and chronic inflammation, may represent a key driver of pro-inflammatory M1 polarization and disease progression. However, existing *in vitro* studies generally suggest that iron overload favors M2 polarization, whereas in the pathological setting of MASLD, iron overload may instead promote M1 polarization. The apparently contradictory effects of iron on macrophage polarization indicate that iron acts as a contextual modulator rather than an autonomous polarizing agent. Its net effect depends on the tissue microenvironment, including coexisting inflammatory cytokines (e.g., IL-6, TNF-α, LPS), the magnitude and duration of iron exposure, and local metabolic conditions such as lipid accumulation and oxidative stress. Under steady-state or anti-inflammatory conditions, iron promotes M2 polarization via NRF2-driven antioxidant responses and suppression of TLR4/NF-κB signaling. In chronically inflamed and lipotoxic environments, however, iron amplifies pro-inflammatory signaling by enhancing IκBα degradation and p65 nuclear translocation, synergizing with lipid peroxidation products to activate the NLRP3 inflammasome and drive M1 polarization (103). Iron overload also induces ferroptosis of resident embryonic Kupffer cells, which are subsequently replaced by monocyte-derived macrophages with a more inflammatory phenotype (105). Thus, the macrophage response to iron is context-dependent. Disease stage, local inflammation, and iron exposure profile should therefore be considered when interpreting the immunometabolic role of iron in macrophage polarization.

Other studies have shown that Fe²^+^ specifically activates NF-κB-dependent pro-inflammatory signaling by promoting IκBα degradation, p65 nuclear translocation, and p50/p65 complex DNA binding, accompanied by ROS generation, and induces TNF-α gene expression and protein release. This mechanism suggests that, in the pathological environment of MASLD, iron loading may directly activate NF-κB and TNF-α signaling, strengthening inflammatory responses and lipid-induced liver injury (105). This study found that iron selectively activates the NF-κB pathway without affecting AP-1 binding or activating JNK, suggesting that designing specific inhibitors targeting iron-mediated NF-κB activation may be more effective than broad-spectrum anti-inflammatory drugs in treating MASLD. Such inhibitors could interrupt inflammatory cascades at the molecular level, thereby halting disease progression while minimizing interference with other essential signaling pathways.

Regarding the pathological driving role of iron homeostasis imbalance in MASLD and MASH on KCs and MoKCs, current basic research and clinical exploration mainly focus on systemic iron removal and chemical iron chelation, targeted iron death inhibition, direct regulation of macrophage populations, as well as nano-targeted delivery and other therapeutic directions. Excessive free iron, through the Fenton reaction, induces strong lipid peroxidation, which directly leads to ferroptosis in liver cells and KCs. In a methionine-choline-deficient (MCD) diet-induced MASH animal model, ferroptosis inhibitors, such as Ferrostatin-1 (Fer-1), Liproxstatin-1 (Lip-1), and vitamin E, neutralize lipid ROS and effectively inhibit ferroptosis, thereby alleviating hepatic inflammation and fibrosis. *In vivo*, Fer-1 and Lip-1 were administered to mice by intraperitoneal injection at 5 mg/kg once daily, while both compounds were used at 1 μM *in vitro*. During treatment, no obvious abnormalities in general behavior, body weight, or liver-to-body weight ratio were observed, and no apparent acute toxicity was reported (100, 106). However, systematic toxicological evaluation, including organ-specific and long-term toxicity assessments, was not performed, limiting conclusions regarding their safety profile. Furthermore, the first two are still experimental tool compounds with no clinical pharmacokinetic or efficacy data. Only vitamin E has been evaluated in human non-alcoholic steatohepatitis (the PIVENS trial), and its mechanism of action is not ferroptosis-specific.

To reduce systemic off-target toxicity, the application of nanocarrier technology has been proposed as a potential therapeutic strategy. By specifically targeting liver or adipose tissue macrophages, researchers potentially utilize superparamagnetic iron oxide nanoparticles (SPIONs) and other nanocarriers for precise drug delivery although such macrophage-targeted applications have not yet been established in metabolic disease (92). For example, SPIONs coated with dimercaptosuccinic acid (DMSA), aminopropyl silane (APS), or aminodextran have been examined in IL-4-activated murine bone marrow-derived M2 macrophages and human THP-1-derived M2-like macrophages. At Fe concentrations of 10–20 μg/mL, these nanoparticles were efficiently internalized, with substantial cell-associated iron detected within 2 h and partial localization in lysosomal/endocytic compartments; importantly, no significant reduction in cell viability was observed even at Fe concentrations up to 100 μg/mL (107). These carriers not only serve as delivery tools but also have the potential to alter intracellular iron metabolism, modulating macrophage activation and potentially favoring some M2-associated features. More inspiringly, SPIONs, as carriers, may influence the metabolic flow of iron within cells, potentially supporting an iron-replete, iron-exporting state in M2 macrophages. This precise targeting of macrophage intracellular metal homeostasis through nanotherapy represents a potential translational direction for the treatment of metabolic liver diseases in the future (107). Because SPIONs can induce ROS-dependent signaling and alter cytokine expression in macrophages, as well as affect other immune-related cell functions, potential off-target immunological effects should be carefully considered.

Multiple studies have demonstrated that patients with MASLD commonly exhibit copper deficiency. Evidence suggests that inadequate copper intake may contribute to the pathogenesis of MASLD. Compared with patients suffering from other liver diseases, individuals with MASLD generally show reduced hepatic and serum copper concentrations (108). Similar findings have been reported in pediatric populations: hepatic copper levels in children with MASLD are significantly lower than those in non-MASLD children, and this difference remains significant even after adjusting for age, BMI, GGT, ALT, and total bilirubin (109). In adult studies, the hair copper concentration of MASLD patients was also found to be markedly lower than that of healthy controls (5). Copper serves as a cofactor for numerous redox enzymes and is essential for maintaining cellular redox homeostasis (110). Dietary fructose can impair intestinal copper absorption, resulting in copper deficiency, which exacerbates oxidative stress and promotes lipid peroxidation in hepatocytes (108). In Jurkat T-lymphocytes, experimentally induced copper deficiency increased cellular susceptibility to H_2_O_2_-induced oxidative DNA damage, indicating that insufficient copper can compromise antioxidant defense capacity. Insufficient copper may therefore weaken cellular antioxidant defenses because copper is an essential cofactor for antioxidant enzymes such as Cu/Zn-SOD and ceruloplasmin. In the hepatic context, such an effect could potentially exacerbate oxidative stress, although this remains to be directly validated in liver macrophages (111). Moderate copper supplementation may represent a low-risk and potentially effective therapeutic approach for MASLD, particularly in pediatric populations (109). Several antioxidant food components known to improve MASLD, including resveratrol, quercetin and its glycoside rutin, oleuropein, curcumin, epigallocatechin-3-gallate (EGCG), luteolin and its derivative luteolin-7-glucoside, as well as caffeic acid and caffeine, possess copper-binding capacity. This observation further supports the potential role of copper as a critical modulator in the prevention and treatment of MASLD (108). In a “copper-deficient + high-fructose diet” (CuMF)–induced MASLD model, KCs were shown to play an essential role. Experimental depletion of KCs completely abolished hepatic triglyceride (TG) accumulation in CuMF-fed rats, accompanied by normalization of plasma monocyte chemoattractant protein-1 (MCP-1) levels and correction of elevated hepatic expression of sterol regulatory element-binding protein-1 (SREBP-1) (99). These findings suggest that KCs may promote hepatic lipid accumulation and inflammation under copper-deficient conditions through MCP-1–mediated inflammatory signaling and SREBP-1–driven lipogenic pathways, serving as a critical regulatory node in MASLD progression.

There is still considerable controversy and a lack of research on the mechanisms regarding the changes in manganese levels in the human body and their effects on diseases, including MASLD. On one hand, large-scale population studies (such as the machine learning analysis based on the UK Biobank) have found that higher manganese intake is closely associated with a lower risk of liver steatosis diagnosed by MRI. From a physiological and biochemical perspective, manganese is a core cofactor of MnSOD and pyruvate carboxylase. Adequate manganese can enhance the liver’s antioxidant defense and maintain normal glucose and lipid metabolism (112). In contrast, some epidemiological and animal studies have found that excessive manganese levels in the blood or liver (manganese overload) are positively correlated with the occurrence of MASLD and abnormal elevation of liver enzymes (such as ALT and AST). Chronic exposure to high concentrations of manganese may cause severe cytotoxicity, disrupt the permeability of the mitochondrial membrane, inhibit the electron transport chain, thereby reducing ATP synthesis and triggering cell necrosis or apoptosis (113). Importantly, elevated circulating manganese levels should not necessarily be interpreted as manganese toxicity or overload. Experimental studies further indicate that excessive and prolonged manganese exposure can cause hepatic manganese accumulation, elevate serum ALT and AST levels, and induce hepatocellular injury (114). The mechanism of manganese’s role in MASLD is still unclear. It may depend on various factors such as the concentration of manganese in the body, the duration of exposure, the source of manganese intake (such as diet or environmental pollution), etc. As a result, the research results show a high degree of variability. Therefore, maintaining a balance of manganese concentration is of significance for liver health.

The accumulation of manganese in the liver microenvironment exerts a strong immunostimulatory effect on macrophages (including resident Kupffer cells and recruited peripheral monocytes), promoting the polarization of macrophages toward an inflammatory phenotype. This process may be related to the following driving mechanisms.

Under conditions of elevated Mn²^+^ availability, manganese can potentiate cGAS–STING signaling and enhance innate immune activation. In porcine alveolar macrophage 3D4/21 cells, Mn²^+^ treatment induces STING- and IRF3-dependent type I interferon signaling, whereas disruption of STING abolishes the Mn²^+^-induced IFN response (115). Importantly, in a ConA-induced autoimmune hepatitis mouse model, manganese overload further activated hepatic cGAS–STING signaling and increased downstream inflammatory mediators, including IFN-α, IFN-β, TNF-α, and IL-6, thereby aggravating liver inflammatory injury (116). however, whether Mn²^+^ specifically amplifies this pathway in hepatic macrophages during MASLD remains to be directly demonstrated. In activated human monocyte-derived macrophages, Mn²^+^ acts as a superoxide dismutase mimetic, increasing H_2_O_2_ formation and thereby altering cellular redox balance (54). In primary mouse macrophages, Mn²^+^ can activate the NLRP3 inflammasome, leading to caspase-1 cleavage and IL-1β release (117). However, whether this Mn²^+^–NLRP3 inflammatory axis operates in Kupffer cells within the lipotoxic microenvironment of MASLD remains to be directly demonstrated. Nevertheless, in IL-4-polarized mouse BMDMs, subsequent exposure to MnCl_2_ (50–400 μM, 24 h) progressively reduced the proportion of M2 macrophages and increased the M1/M2 ratio, as assessed by flow cytometry. Mn²^+^ treatment also increased CD80 expression and IFN-β secretion and enhanced cGAS–STING–IRF3/NF-κB signaling (118). These findings suggest that elevated Mn²^+^ availability can promote inflammatory macrophage repolarization, although whether a similar mechanism contributes to hepatic macrophage activation in MASLD remains unknown.

Most of the current *in vitro* mechanism studies on metal immunology rely heavily on bone marrow-derived macrophages or monocyte macrophage lines as model systems. This approach has advantages in terms of obtaining cell yields and experimental reproducibility, but it ignores the complex cell heterogeneity and masks the essential differences in manganese overload responses between embryonic-originating resident KCs and MoMFs. For instance, whether the tolerance thresholds of Kupffer cells to manganese toxicity and cGAS-STING activation are fundamentally different from those of newly recruited macrophages. Solving such problems will provide crucial clues to deeply understand the immune mechanisms in the context of manganese overload. Despite these preliminary findings demonstrating that manganese influences inflammation and immune status through macrophages, direct evidence in the context of MASLD remains limited. The mechanistic effects of manganese on KCs lack systematic *in vivo* and clinical validation, as most conclusions are derived from *in vitro* macrophage models or tumor and arthritis models, which cannot fully reflect cell behavior in the liver-specific microenvironment. Future studies could also integrate clinical cohort analyses to examine the associations among serum manganese levels, hepatic inflammatory markers, and MASLD staging, aiming to identify potential early intervention biomarkers.

Studies have shown that zinc can prevent the translocation of endotoxins across the intestinal barrier, thereby reducing alcohol-induced liver injury. Zinc pretreatment inhibits LPS-induced expression and activation of TNF-α and NF-κB in KCs, providing partial protection to these cells (119–121). Bacterial stimulation can induce KCs to secrete thromboxane (TX) A2, accompanied by upregulation of MyD88, MAPK, and NF-κB gene expression, which exacerbates hepatocyte injury and chronic inflammation, whereas zinc pretreatment can alleviate the inflammatory burden (120). It is worth noting that the protective effects of zinc are cell type–specific, primarily targeting KCs, while having limited impact on other non-parenchymal liver cells. Further studies have shown that zinc suppresses TNF-α production by inhibiting the oxidative stress–sensitive NF-κB signaling pathway (121), and this anti-inflammatory effect may help alleviate liver inflammation associated with MASLD. However, these studies primarily focus on acute bacterial infection or LPS-induced liver injury models, and whether the protective effects of zinc observed in these acute inflammatory settings are applicable to the chronic progression of MASLD remains to be further validated.

**Table 2** comprehensively summarizes the roles of various molecular targets and signaling pathways in regulating macrophage function and further outlines their impact on the progression of MASLD. By integrating pathways related to inflammatory signaling, oxidative stress, lipid metabolism, and immune regulation, It is becoming increasingly apparent that macrophages occupy a central role within the chronic inflammatory microenvironment of the liver.

**Table 2:** Transition metal-mediated macrophage targets and signaling pathways in MASLD pathogenesis.

Metals	Target (Pathway/functional node)	Effect on macrophage	Effects on MASLD	Authors	Evidence source
Fe	Hepcidin–FPN axis	Chronic inflammation and leptin upregulate hepcidin, promoting FPN degradation on KCs and causing macrophage iron retention. In some MASH models, reduced hepcidin may instead increase systemic iron availability.	Causes hepatic iron dysregulation and contributes to DIOS. Increased hepcidin promotes local hepatic iron overload, whereas hepcidin deficiency can enhance intestinal iron absorption and lead to systemic iron overload.	98;	Animal
	TFEB/Ferritin Autophagy	Iron overload is proposed to increase intracellular Ca²^+^–calcineurin signaling, promoting TFEB nuclear translocation and ferritinophagy, which enhances iron-dependent oxidative stress and hepatic ferroptosis	Amplifies oxidative stress and lipid peroxidation. The loss of resident KCs weakens the liver’s anti-inflammatory barrier and triggers damage-associated signals.	100;	Animal
	Fe–MiT/TFE axis	Iron accumulation activates MiT/TFE transcription factors and drives Fe-hi KCs toward pro-inflammatory and pro-fibrotic phenotypes.	Promotes hepatic inflammation and fibrosis in MASH; iron chelation attenuates fibrosis.	102	Animal + primary-cell + cell-line + human tissue
	SPION–ROS–ERK/AKT–M2 remodeling	SPION uptake by M2 macrophages increases ROS and activates ERK/AKT signaling, shifts intracellular iron metabolism toward an iron-replete state, and alters M2-associated functional programs.	Provides a potential macrophage-targeted strategy for manipulating intracellular iron handling, but its therapeutic efficacy and immunological effects in MASLD remain unvalidated.	107	Primary-cell + cell-line + nanoparticle
	ROS/NF-κB Pathway	Fe²^+^ specifically promotes IκBα degradation and p65 nuclear translocation. This binds DNA and activates the transcription of pro-inflammatory cytokines like TNF-α.	Selectively amplifies NF-κB–TNF-α signaling without activating other pathways (e.g., AP-1 or JNK), strengthening persistent lipid-induced liver injury.	105	Primary-cell
Cu	Low-Copper Microenvironment	A copper-deficient state disrupts iron efflux processes. Even if KCs are depleted, this microenvironment prevents the clearance of iron loads.	Leads to systemic iron metabolic dysregulation and abnormal iron deposition primarily in hepatocytes, sustaining overall liver injury and limiting the efficacy of KC-targeted therapies.	99	Animal
	Cellular redox homeostasis/Antioxidant defense	Insufficient copper compromises cellular antioxidant defense systems (copper serves as a vital cofactor for redox enzymes).	Potentially promotes hepatic inflammation and fibrosis in MASH; iron chelation attenuates fibrosis.	110 108	Animal (Tallino)
	MCP-1 and SREBP-1 pathways	Under copper-deficient conditions, Kupffer cells (KCs) are activated to mediate inflammatory signaling via monocyte chemoattractant protein-1 (MCP-1) and drive lipogenic pathways via SREBP-1.	Promotes massive hepatic triglyceride (TG) accumulation and inflammation. Depleting KCs completely abolishes these changes, indicating KCs are a critical regulatory node in disease progression.	99	Animal
Zn	Zn–ROS/NF-κB–TNF-α axis	Zinc pretreatment suppresses LPS-induced superoxide generation and NF-κB activation in KCs, thereby reducing TNF-α production.	May attenuate hepatic inflammatory injury; however, the evidence derives from acute LPS-induced liver injury rather than chronic MASLD.	121	Animal
	Zn–MyD88/MAPK/NF-κB–TXA_2_ axis	In human KCs, zinc pretreatment suppresses bacteria-induced MyD88, MAPK and NF-κB expression and reduces TXA_2_ secretion and cellular injury.	Suggests a KC-specific anti-inflammatory role of zinc, although direct validation during chronic MASLD progression is lacking.	120	Human + primary-cell + cell-line
Mn	MnSOD and Pyruvate Carboxylase	Adequate Mn acts as a core cofactor to enhance cellular antioxidant defenses and maintain normal metabolic functions.	Protective effect: Maintains normal glucose/lipid metabolism and is associated with a lower risk of MRI-diagnosed liver steatosis.	112	Human population-based association
	Mn²^+^–cGAS–STING–IRF3 axis	Activates STING–IRF3-dependent type I IFN signaling and enhances inflammatory responses.	Potentially aggravates hepatic inflammation; direct validation in MASLD macrophages is still lacking	115; 116	Cell-line (Zheng) Animal (Saimaier)
	ROS/NLRP3 Inflammasome	Mn²^+^ increases H_2_O_2_ formation in activated macrophages, altering cellular redox balance. In primary mouse macrophages, Mn²^+^ activates the NLRP3 inflammasome, promoting caspase-1 cleavage and IL-1β release.	May amplify oxidative and inflammasome-associated inflammation in MASLD; however, direct validation of this pathway in hepatic macrophages during MASLD remains lacking.	54; 117	Primary-cell
	Interferon Signaling/M2-to-M1 Reversal	Mn²^+^ reduces M2 macrophage proportions, increases the M1/M2 ratio, and enhances CD80, IFN-β and cGAS–STING–IRF3/NF-κB signaling.	Potentially enhances inflammatory macrophage responses; direct evidence in MASLD hepatic macrophages is lacking.	118	Primary-cell + cell-line

cGAS, cyclic GMP-AMP synthase; MASLD, metabolic dysfunction-associated steatotic liver disease; MCP-1, monocyte chemoattractant protein-1; MnSOD, manganese superoxide dismutase; NF-κB, nuclear factor kappa light chain enhancer of activated B cells; NLRP3, NOD-, LRR- and pyrin domain-containing protein 3; ROS, reactive oxygen species; SREBP-1, sterol regulatory element-binding protein-1; STING, stimulator of interferon genes; TFEB, transcription factor EB; TNF-α, tumor necrosis factor-alpha.

### 3.3 Metal-induced immune dysregulation in AS

Worldwide, cardiovascular disease is a leading cause of mortality, responsible for an estimated 16.7 million deaths annually, with atherosclerosis being the primary underlying cause of cardiovascular disease (28, 120). AS is a common inflammatory vascular disease characterized by plaque formation, mainly comprising lipid-carrying foam cells, and driven by endothelial cell activation, increased oxidative stress, and immune cell dysfunction (89, 122, 123). The development of AS begins with the accumulation of LDL in the arterial intima. Apolipoprotein B100 can bind to proteoglycans in the extracellular matrix via ionic interactions, leading to subendothelial retention of LDL. This retention is regarded as a key initiating event in the early development of AS. AS is recognized as an inflammatory disorder involving both the innate and adaptive branches of the immune system. LDL, particularly the abundance of apolipoprotein B, is closely associated with human AS and is therefore a candidate antigen for vaccine development (124). The retained LDL is prone to enzymatic oxidation in the intima or attack by ROS generated during inflammation and AS, such as HOCl, phenoxyl radical intermediates, and peroxynitrite cause peroxidation of fatty acid chains in phospholipids, cholesteryl esters, and triglycerides, producing reactive aldehydes and truncated lipid molecules (123, 125). Components of ox-LDL activate endothelial cells, inducing the expression of adhesion molecules such as E-selectin and vascular cell adhesion molecule-1 (VCAM-1) on the arterial endothelium. In conjunction with chemokines including CCL2, CCL5, CXCL10, and CX3CL1, these signals facilitate the recruitment of monocytes, dendritic cells, and T cells into the intima. Once in the intima, monocytes differentiate into macrophages in response to macrophage colony-stimulating factors secreted by activated endothelial cells, representing a crucial step in the initiation and progression of AS (123).

Macrophages within atherosclerotic lesions are primarily derived from resident arterial macrophages and recruited circulating monocytes, and their relative contributions change with disease progression. Resident arterial intimal macrophages (MacAIR) are maintained mainly by local proliferation under physiological conditions. at the onset of hypercholesterolemia, they are among the first cells to take up lipids and form foam cells, thereby contributing to lesion initiation. As lipid accumulation and inflammation persist, circulating monocytes are recruited into lesions through pathways involving CCR2, CCR5 and CX3CR1 and subsequently differentiate into MoMFs (126). As the disease advances, the relative contribution of the original MacAIR population gradually declines, whereas MoMFs become the predominant macrophage population within established plaques (126). After monocytes infiltrate plaques, they develop into diverse macrophage states under the stimulation of microenvironmental cues such as ox-LDL, hypoxia and inflammatory factors, including inflammatory non-foam macrophages, TREM2^+^ lipid-associated macrophages, and interferon-inducible macrophages (126, 127). Among these subsets, inflammatory macrophages predominantly express proinflammatory genes, whereas TREM2^+^ lipid-associated macrophages display active programs of lipid metabolism, oxidative phosphorylation, cholesterol efflux and efferocytosis (127). In addition, microenvironment-induced macrophage states such as Mox, M4 and Mhem/M(Hb) also exist in plaques, which are triggered by oxidized phospholipids, CXCL4, and hemoglobin/heme stimulation, respectively (75). Collectively, plaque macrophages exhibit substantial heterogeneity in origin and function. Therefore, the conventional M1/M2 binary classification cannot fully capture the complexity of macrophages in atherosclerotic plaques.

Among them, the abnormal distribution, metabolism and transportation of iron in the body are particularly significant. The main manifestation is severe iron overload in the local tissues and the core, such as in macrophages. This pathological iron overload is primarily driven by iron deposition in the local microenvironment and late plaque hemorrhage. Numerous clinical and histopathological studies have shown that iron concentrations in human atherosclerotic plaques are significantly higher than those in healthy arterial walls (32). As the AS lesion progresses, a large number of structurally fragile and highly permeable new neovessels tend to form within the plaque. neovessels are highly susceptible to rupture, thereby causing intraplaque hemorrhage (128). During this process, a large number of red blood cells enter the core of the plaque and undergo dissolution, releasing the abundant hemoglobin and free iron with high redox activity directly into the microenvironment of the plaque, constituting the direct physical source of a sharp increase in iron concentration in the lesion area (129). Additionally, the AS microenvironment induces uncontrolled iron uptake and retention by macrophages. Under the continuous stimulation of pro-inflammatory cytokines and oxidized modified ox-LDL and other pathogenic factors, the expression of Transferrin receptor protein 1 (TfR1) on the surface of macrophages is significantly upregulated (130). This increased receptor expression drives excessive iron uptake from the surrounding microenvironment. At the same time, to buffer the sharp increase in intracellular iron toxicity, ferritin expression is compensatorily upregulated, promoting sustained intracellular iron sequestration and accumulation (130). However, the most fundamental mechanism that exacerbates the accumulation of iron within macrophages lies in the blockade of iron excretion mediated by the hepcidin-FPN axis. Under the chronic inflammatory state of AS, the levels of hepcidin, which is secreted systemically by the liver or locally secreted by macrophages in the plaque, significantly increase (128, 130). To sum up, The influx of exogenous iron due to pathological microvascular rupture, the uncontrolled intake caused by excessive expression of TfR1 receptors, and the blockade of the FPN excretion pathway caused by hepcidin all jointly promote iron overload. Therefore, plaque hemorrhage, hepcidin-mediated FPN degradation, iron retention, ROS generation, and foam cell formation constitute a self-amplifying metal–macrophage feedback loop that drives plaque instability.

Disordered iron metabolism profoundly alters macrophage morphology and function through multiple biochemical pathways, including oxidative stress, phenotypic polarization, and cell death, thereby promoting foam cell formation. First, as a redox-active transition metal, excess Fe²^+^ catalyzes the Fenton reaction (130). This not only directly oxidatively modifies LDL into pro-atherogenic ox-LDL but also leads to substantial intracellular production of ROS in macrophages (129). Such iron overload–induced oxidative stress significantly downregulates the transcription factor LXRα, thereby suppressing the expression of ATP-binding cassette (ABC) transporters responsible for cholesterol efflux. Consequently, macrophages fail to effectively export excess lipids, leading to intracellular cholesterol accumulation and accelerated foam cell formation (32). In addition, iron promotes nuclear translocation and activation of 5-LOX in macrophages, enhancing the production of leukotrienes and pro-inflammatory cytokines such as IL-6 (131). When intracellular iron overload combines with severe lipid peroxidation, it triggers ferroptosis, an iron-dependent form of programmed cell death, leading to massive macrophage rupture. This ferroptosis-associated oxidative stress not only causes cellular injury but also impairs lipid efflux capacity, resulting in uncontrolled lipid accumulation and further accelerating foam cell transformation (132).

In traditional theories of atherosclerosis, macrophages are typically classified into pro-inflammatory M1 and anti-inflammatory M2 phenotypes. However, with the development of single-cell RNA sequencing technology, researchers have identified a highly specialized subset of macrophages in atherosclerotic plaques. These cells express high levels of a transmembrane lipid-sensing receptor called TREM2 on their surface. Contrary to traditional understanding, these lipid-rich cells do not exhibit strong pro-inflammatory features. Instead, they initiate a gene expression program associated with tissue homeostasis, lipid metabolism, lysosomal degradation, and efferocytosis (the clearance of apoptotic cells). As a result, TREM2^+^ macrophages are considered the scavengers in the plaque microenvironment, attempting to maintain local metabolic and immune balance under the extreme pressure of lipid overload (126, 133). In advanced atherosclerotic plaques, rupture of newly formed microvessels often leads to intraplaque hemorrhage, releasing large amounts of aged red blood cells, hemoglobin, and highly toxic free iron (128). In this extreme microenvironment, TREM2 not only senses lipids but also indirectly and critically regulates macrophage buffering capacity against iron toxicity. Mechanistically, TREM2 signaling maintains and upregulates the expression of HO-1 in macrophages. A principal function of HO-1 is to degrade heme and safely process the released iron. When macrophages lack TREM2, the transcriptional levels of both HO-1 and the anti-apoptotic gene Bcl2 are significantly reduced (128, 134). In addition, TREM2 deficiency is accompanied by elevated ROS, increased lipid peroxidation and aggravated tissue injury or death (135). Therefore, we hypothesize that TREM2 exerts a protective effect on plaque macrophages by maintaining HMOX1-dependent heme catabolism and broader iron/redox homeostasis. Loss of TREM2 impairs this protective mechanism, rendering macrophages more susceptible to iron-mediated reactive oxygen species accumulation, lipid peroxidation, and cell death. Under the combined burden of iron toxicity and lipid overload, macrophages undergo extensive cell death. Together with impaired efferocytosis, this process promotes the formation of a large necrotic core that is highly susceptible to rupture within the plaque (134, 136). In contrast, the use of TREM2 agonists such as the antibody 4D9 can enhance macrophage survival and clearance abilities, significantly reducing the necrotic core, increasing plaque collagen deposition, and greatly improving plaque stability (127).

Targeting macrophage ferroptosis has emerged as a promising strategy for stabilizing vulnerable plaques. Upregulation of the core anti-ferroptosis enzyme glutathione peroxidase 4 (GPX4) or the use of free radical scavenging antioxidants can interrupt lipid peroxidation cascades, thereby preventing rapid expansion of the necrotic core (132). Recent studies further demonstrate that the ferroptosis inhibitor ferrostatin-1 activates the AMP-activated protein kinase (AMPK) pathway in macrophages, coordinately upregulating ferritin heavy chain (FTH), GPX4, and scavenger receptor class B member 1 (SCARB1), and significantly reducing iron overload and lipid accumulation in ox-LDL-treated macrophages both *in vivo* and *in vitro* (132). However, a key limitation is that the precise transcription factors, upstream enzymes, or metabolic bypasses through which AMPK regulates these molecules remain unclear. Elucidating this causal network would represent a breakthrough in AS therapy. As discussed above, copper serves as an essential enzymatic cofactor in macrophages, including for copper-dependent enzymes such as ceruloplasmin, and macrophage copper homeostasis is dynamically regulated by transporters such as CTR1 and ATP7A during inflammatory activation (35). Copper also exhibits bidirectional regulation of inflammatory responses: at higher concentrations, it can activate NF-κB, MAPKs, and the NLRP3 inflammasome, promoting the release of pro-inflammatory cytokines such as IL-1β, IL-6, and TNF-α; whereas at lower concentrations, it promotes macrophage polarization toward the M2 phenotype, thereby exerting anti-inflammatory effects and supporting tissue repair (37).

In the pathological context of AS, the concentration and distribution of endogenous copper are significantly remodeled. Clinical and animal model studies consistently show that copper content in atherosclerotic lesions is markedly higher than in healthy vascular walls, and this local copper accumulation is positively correlated with the severity of vascular inflammation (133). Although direct *in vivo* evidence demonstrating that disrupted copper homeostasis alone initiates AS progression remains lacking, existing findings in macrophage molecular biology suggest that elevated endogenous copper levels may accelerate disease progression through several mechanisms. First, abnormally released free copper within plaques can participate in redox cycling reactions and generate highly destructive hydroxyl radicals through Fenton chemistry (137). These radicals induce strong lipid peroxidation, directly oxidizing LDL into pathogenic ox-LDL (35, 137). Macrophages subsequently take up large amounts of ox-LDL via scavenger receptors, ultimately leading to excessive cholesterol accumulation and foam cell formation. The previously described CTR1–ATP7A copper-trafficking system may be particularly relevant to this process in plaque macrophages. Notably ATP7A is expressed in macrophages within atherosclerotic lesions and promotes macrophage-mediated LDL oxidation, at least partly through regulation of cPLA_2_α activity (138).Collectively, these findings suggest that CTR1–ATP7A-dependent copper trafficking may contribute to oxidative lipid processing in plaque macrophages, potentially linking altered intracellular copper handling to LDL oxidation and foam cell formation, although the precise subcellular link to LDL oxidation remains unclear.

Copper accumulation may also amplify inflammatory signaling and macrophage death within lesions. In copper-exposed BV2 microglial cells, intracellular copper accumulation induces ROS-dependent NF-κB activation and promotes the production of pro-inflammatory mediators such as TNF-α and IL-6. However, this study was conducted in a neuroinflammatory model rather than an atherosclerotic setting, so whether the same copper-responsive pathway operates in plaque macrophages remains to be directly established (137, 139). In addition, copper ions can destabilize lysosomes and directly mediate activation of the NLRP3 inflammasome, thereby resulting caspase-1–dependent pyroptosis and the release of IL-18 and IL-1β (J. 133). Abnormal macrophage death within plaques and impaired efferocytosis are key contributors to necrotic core formation and plaque rupture (75). Moreover, massive death and rupture of foam cells release lipid cores and pro-inflammatory mediators into the plaque, accelerating necrotic expansion and leading to plaque instability.

Given the complex pathogenesis of AS, current therapeutic strategies may focus on regulating copper metabolism, modulating intracellular copper levels in macrophages, influencing macrophage polarization, and inhibiting pathological cell death. Copper chelators such as tetrathiomolybdate (TTM) and trientine have been widely used in Wilson’s disease (copper overload). In preclinical studies of AS, TTM has been shown to effectively reduce plaque burden in mice. Its mechanisms include lowering bioavailable labile copper levels, inhibiting NF-κB and activator protein-1 (AP-1) signaling pathways, and thereby reducing the expression of VCAM-1, intercellular adhesion molecule-1 (ICAM-1), and inflammatory cytokines, ultimately slowing plaque progression. The broad-spectrum chelator ethylenediaminetetraacetic acid (EDTA) has also been reported to reduce cardiovascular event risk (137). In advanced plaques, impaired macrophage efferocytosis leads to defective clearance of apoptotic cells, resulting in secondary necrosis and plaque instability. Targeting the overexpression of pro-resolving lipid mediator receptors such as G protein-coupled receptor 32 (GPR32) can enhance inflammation resolution, significantly reduce lesion size, and decrease intraplaque necrosis (75). Another promising strategy is to restore cellular copper homeostasis by targeting ATP7A trafficking. Activation of protein kinase B isoform 2 (Akt2) signaling or the use of small-molecule chaperones to stabilize ATP7A and promote its translocation to the plasma membrane can enhance cellular copper efflux. This restores autonomous copper regulation in macrophages, enabling efficient copper export via membrane-localized ATP7A, thereby strengthening the fibrous cap without disturbing systemic copper homeostasis (140).

Frontier therapeutic strategies for AS increasingly focus on nanotechnology, aiming to simultaneously intervene in key pathological processes such as macrophage phagocytosis, lipid degradation, and cholesterol efflux to achieve plaque stabilization and inflammation reduction. Recently, a hollow mesoporous manganese dioxide nanomedicine coated with a macrophage membrane (Col@HMnO_2_-MM) was developed. In ApoE^-^/^-^ atherosclerotic mice, DiR-labeled Col@HMnO_2_-MM was administered intravenously at 3 mg/kg for targeting and biodistribution studies, while therapeutic administration was performed twice weekly for 8 weeks. The nanomedicine accumulated in atherosclerotic lesions, eliminated ROS to alleviate the inflammatory storm, improved local hypoxia, and downregulated HIF-1α, thereby enhancing cholesterol efflux and reducing foam cell formation. Biodistribution analysis showed prominent accumulation in the liver and spleen. Initial safety evaluations, including cell viability, hemolysis, hematological and biochemical parameters, and histological examination of major organs, revealed no obvious abnormalities under the tested conditions. Nevertheless, these findings are limited to cell and ApoE^-^/^-^ mouse models, and potential long-term manganese toxicity, interactions with endogenous metals such as Fe, Cu, and Zn, as well as broader off-target effects remain insufficiently characterized. Thus, further pharmacokinetic, toxicological, and translational studies are required before clinical application (141). CEZP nanoparticles integrate CpG, Zn²^+^, and EGCG, can also accumulate within atherosclerotic plaques and promote macrophage uptake, thereby exerting a multifunctional therapeutic effect through complementary mechanisms. Specifically, Zn²^+^ enhances macrophage lipid degradation by promoting autophagy, reduces intracellular lipid burden, and attenuates foam cell formation. In addition, CEZP treatment was associated with reduced inflammatory signaling and a shift in macrophage markers from an M1-like toward an M2-like phenotype, thereby intervening in AS pathology at multiple levels (142).

*In vivo* experiments have shown that CEZP significantly reduces plaque lipid burden and improves indices associated with plaque stability. In ApoE^-^/^-^ mice, CEZP was administered by tail-vein injection at 15 mg/kg every 5 days for 10 doses; equivalent free-component doses were CpG 63.6 nmol/kg, EGCG 5.86 mg/kg, and Zn²^+^ 1.45 mg/kg. Biodistribution studies showed that CEZP was not restricted to plaques and was also detected in major organs, with liver and kidney being important sites of distribution and elimination. No overt short-term hepatic, renal, hematological, or major-organ histopathological toxicity was detected under the tested conditions, although these assessments do not establish long-term safety. This study shows that CpG enhances efferocytosis, Zn²^+^ promotes intracellular lipid degradation, and EGCG upregulates ATP-binding cassette transporters to facilitate cholesterol efflux. However, the mechanistic interdependence and temporal coordination among these three processes were not systematically resolved. Although replacement of Zn²^+^ with Cu²^+^ abolished the lipid-degrading effect, the study did not report systematic evaluation of interactions between released Zn²^+^ and endogenous metal-ion homeostasis. Moreover, despite evidence of CpG–TLR9 engagement, systemic off-target immunological effects were not comprehensively assessed. The nanoparticle contained EGCG and Zn²^+^ at 39.09% and 9.66%, respectively; however, whether the therapeutic dose ratio among CpG, Zn²^+^, and EGCG is optimal remains unclear, because the reported formulation optimization mainly addressed MOF formation rather than comparative therapeutic efficacy across different component ratios. The study itself provides no human interventional data for CEZP, so its therapeutic efficacy and safety remain supported primarily by preclinical evidence (142).

In diagnostics, strategies leveraging nanotechnology and metal regulation also show cutting-edge application potential. Researchers developed ultrasmall zinc ferrite (Zn_0_._4_Fe_2_._6_O_4_) nanoparticles, whose surface charge is modified to enhance macrophage uptake. Positively charged nanoparticles exploit the negative charge of macrophage membranes to significantly improve T1-weighted MRI contrast and signal intensity for AS plaques while maintaining low toxicity and good biocompatibility. Metal components play key roles: Zn²^+^ enhances nanoparticle electrostatic interactions with macrophages, increasing uptake efficiency and providing functional support for MRI; Fe³^+^ serves as the magnetic core, amplifying imaging signals for precise plaque localization. This strategy offers an innovative approach for monitoring macrophage function and dynamic imaging of AS plaques (143). Flow cytometry analysis of rabbit peritoneal macrophages showed that positively charged Zn_0_._4_Fe_2_._6_O_4_-NH_2_ nanoparticles exhibited the highest cellular uptake, attributed to electrostatic interaction with negatively charged macrophage membranes. The potential limitations of this non-specific electrostatic interaction remain to be demonstrated. While positively charged nanoparticles enhance uptake efficiency, their lack of specificity may lead to non-specific binding with other negatively charged cells. Future studies could consider introducing macrophage-specific targeting ligands, such as CD68, CD163, or mannose receptor surface markers, to achieve precise targeting of macrophages and improve imaging effectiveness.

Transition metal–dependent alterations in macrophage function influence the intraplaque inflammatory microenvironment, lipid burden, and plaque stability, thereby collectively promoting or alleviating the progression of atherosclerosis. **Table 3** summarizes the principal targets and pathways of different transition metals in macrophages, as well as their effects on atherosclerosis progression.

**Table 3:** Transition metal-mediated macrophage targets and signaling pathways in atherosclerosis pathogenesis.

Metal	Target (pathway/node)	Effect on macrophage	Authors	Evidence source of the pathway
Fe	Hepcidin-FPN	Hepcidin degrades FPN. Iron is trapped intracellularly.	32;	Animal
	Fenton reaction & LXRα-ABC	Fe²^+^ creates ROS. Oxidative stress halts lipid efflux by suppressing LXRα and ABC transporters.	129; 32	Animal (Cai)
	5-LOX pathway	Iron promotes 5-LOX activation and its association with FLAP, favoring leukotriene biosynthesis and enhancing pro-inflammatory IL-6 production.	131	Primary-cell
	Ferroptosis	Iron overload and lipid peroxidation promote ferroptosis, potentially impairing macrophage cholesterol efflux through dysregulation of GPX4–SCARB1-related lipid metabolism.	132	Animal + cell-line
	Trem2/Hmox1 axis	TREM2 may facilitate macrophage adaptation to the iron/heme-rich niche in advanced plaques via HMOX1-dependent heme catabolism. TREM2 deficiency may impair this protection, increasing macrophage vulnerability to iron-mediated oxidative injury and cell death.	126; 133; 128; 134; 135	Animal + primary- cell (Piollet) animal + primary-cell (Perugorria)
	AMPK, GPX4, FSP1, DHODH	AMPK upregulates FTH/GPX4. FSP1 and DHODH inhibit macrophage ferroptosis independently.	132;	Animal + cell-line
Cu	Ceruloplasmin Activity	Cu acts as a cofactor for Cp ferroxidase, converting Fe²^+^ to Fe³^+^ for safe iron export.	35; 36	Cell-line (White) primary-cell + cell-line (Jonczy)
	CTR1-ATP7A axis	Inflammatory remodeling of the CTR1–ATP7A axis may increase macrophage copper uptake and redirect ATP7A to vesicular compartments, thereby promoting LDL oxidation and foam-cell formation.	35; 138	Cell-line (White) Animal + cell-line (Qin)
	NF-κB, & NLRP3 Pathways	Copper accumulation in BV2 microglia triggers ROS-dependent NF-κB activation and secretion of proinflammatory mediators including TNF-α and IL-6.	139 137	Animal + cell-line (Zhou)
	Cu–ROS/Fenton–ox-LDL axis	Redox-active Cu promotes ROS generation and lipid peroxidation, facilitating LDL oxidation to ox-LDL and subsequent macrophage foam-cell formation.	137; 35	Cell-line (White)
	Akt2–ATP7A–Cu efflux axis	Akt2-dependent ATP7A trafficking to the plasma membrane promotes cellular Cu efflux and may restore macrophage copper homeostasis.	140	Animal + primary-cell

5-LOX, 5-lipoxygenase; ABC, ATP-binding cassette; AMPK, AMP-activated protein kinase; ATP7A, ATPase copper transporting alpha; CTR1, copper transporter 1; DHODH, dihydroorotate dehydrogenase; DLAT, dihydrolipoamide S-acetyltransferase; FPN, ferroportin; FSP1, ferroptosis suppressor protein 1; FTH, ferritin heavy chain; GPX4, glutathione peroxidase 4; Hmox1, heme oxygenase 1; IL-1β, interleukin-1 beta; IL-6, interleukin-6; LDL, low-density lipoprotein; LXRα, liver X receptor alpha; MAPK, mitogen-activated protein kinase; NF-κB, nuclear factor kappa B; NLRP3, NLR family pyrin domain containing 3; ox-LDL, oxidized low-density lipoprotein; ROS, reactive oxygen species; TCA, tricarboxylic acid; TfR1, transferrin receptor 1; TNF-α, tumor necrosis factor alpha; Trem2, triggering receptor expressed on myeloid cells 2.

## 4 Limitations

Several examples discussed in this review indicate that some of the current inferences extend beyond what can be supported by direct causal evidence. Although experimental interventions in specific models can establish causal relationships for individual readouts, a complete causal chain linking metal dyshomeostasis, macrophage dysfunction, and human metabolic diseases has not yet been demonstrated. In particular, changes in cytokine levels, ROS, or M1/M2-related markers alone are insufficient to demonstrate corresponding alterations in overall macrophage function, nor do they establish that macrophages necessarily mediate changes in disease outcomes such as glycemic control, liver injury, or AS plaque burden.

Iron provides a representative example. Experimental studies have frequently associated iron retention with an M2-like phenotype; however, in lipid-rich and chronically inflamed microenvironments such as those found in MASLD or AS plaques, iron accumulation may instead be accompanied by M1-like activation, oxidative stress, foam-cell formation, or ferroptotic injury. In diabetic models, long-term high-iron diets may even exert context-dependent protective effects in certain tissues. These apparently contradictory findings indicate that iron does not exert a uniform or fixed direction of effect, and that the tissue microenvironment, disease stage, macrophage subset, as well as the chemical form and subcellular distribution of iron, may alter its biological effects. Therefore, associations between iron levels and polarization markers alone are insufficient to conclude that iron dyshomeostasis drives disease progression through a specific macrophage phenotype. Ferrostatin-1 can reduce iron burden and lipid accumulation in ox-LDL-treated macrophages and involves AMPK, FTH, GPX4, and SCARB1; however, the specific regulatory network through which AMPK controls these downstream molecules remains unclear. In MASLD-related studies, the protective effects of ferroptosis inhibitors such as Ferrostatin-1 and Liproxstatin-1 are derived mainly from MASH animal models and *in vitro* experiments, while organ-specific and long-term toxicological data remain lacking, thereby limiting safety assessment. These compounds remain experimental tools and lack clinical pharmacokinetic and efficacy data. In MASLD, EmKCs and MoKCs differ markedly in their origin, function, and inflammatory phenotype, whereas it remains unclear whether they exhibit similar tolerance thresholds, uptake capacities, and stress responses to metals such as iron and manganese.

For zinc, zinc supplementation trials have generally shown limited metabolic benefits with substantial interindividual variability, and there is currently insufficient evidence to demonstrate that the potential benefits in humans are mediated by restoration of macrophage function. Existing ZnT8 knockout studies have established the importance of ZnT8 and zinc for the formation of insulin dense-core granules, but this does not directly demonstrate that β-cell failure in typical patients with T2DM is primarily caused by zinc deficiency or impaired ZnT8 function. Similarly, the hypothesis that Zn(Mal)_2_ attenuates macrophage inflammation by suppressing the release of FFAs from adipocytes has a certain degree of biological plausibility. Previous studies suggest that Zn(Mal)_2_ can regulate adipocyte lipolysis and metabolism, while adipocyte-derived FFAs can mediate inflammatory crosstalk between adipocytes and macrophages. However, direct experimental evidence demonstrating that Zn(Mal)_2_ regulates macrophage polarization, recruitment, or tissue infiltration through this pathway is still lacking. Most mechanistic studies employ short-term metal stimulation, LPS intervention, or *in vitro* cell exposure, whereas T2DM, MASLD, and AS are all chronic progressive diseases. For example, evidence that zinc suppresses NF-κB/TNF-α signaling in KCs is derived mainly from acute LPS or infection models, and whether this effect is applicable to the chronic lipotoxic and low-grade inflammatory microenvironment of MASLD has not yet been demonstrated. For copper, direct *in vivo* evidence showing that copper dyshomeostasis alone is sufficient to induce AS remains lacking. Therefore, the associations among copper accumulation, LDL oxidation, inflammasome activation, macrophage death, and plaque instability can only be regarded as a mechanistic framework supported by multiple mutually reinforcing but still incomplete lines of evidence. In particular, extrapolating ATP7A intracellular trafficking and copper redistribution observed during infection or antimicrobial responses to a copper-dependent LDL oxidation mechanism within AS plaques still requires direct validation in the plaque-specific microenvironment. Whether copper transport regulation observed under infectious stimulation also occurs in the lipid-rich, hypoxic, and chronically inflamed plaque microenvironment, and whether ATP7A is required for macrophage-mediated plaque progression, remain to be further established. Evidence linking copper to MASLD is currently limited to observational associations: several cross-sectional studies have reported reduced copper levels in the liver, serum, or hair of patients with MASLD, but these studies cannot determine whether copper deficiency is a cause of the disease or a secondary consequence. At the same time, mechanistic conclusions regarding copper regulation of macrophage function are largely derived from models of copper imbalance or LPS/IFN-γ stimulation and therefore do not fully represent physiological homeostasis in humans.

For cobalt, the hypothesis that B12 deficiency leads to abnormal methylmalonyl-CoA metabolism, reduced succinyl-CoA availability, impaired OXPHOS, and subsequently promotes a shift of macrophages toward an M1-like state has not yet been established as a complete causal chain. Current studies support only associations among several individual components of this proposed pathway.

The conclusions regarding manganese are even more inferential. Population studies suggest that higher dietary manganese intake is associated with a lower risk of hepatic steatosis, whereas other epidemiological and animal studies suggest that manganese overload is associated with MASLD and elevated liver enzymes. These apparently contradictory findings make it difficult to determine whether the discrepancies arise from differences in exposure duration or from the complex physiological context created by interactions between metal homeostasis and metabolism in humans.

The efficacy of metal-based nanotherapies is influenced by particle size, surface charge, coating, and distribution in non-macrophage tissues. As a multicomponent system, CEZP contains CpG, zinc ions, and EGCG, which act independently on different pathways; however, the mechanistic synergy among these components, the necessity of each component, and the optimal ratio have not yet been clarified. CEZP was evaluated using a fixed dose and a limited treatment period. Although no obvious short-term toxicity was observed, its long-term safety remains uncertain; therefore, short-term efficacy in animal studies cannot be directly equated with sustained safety and efficacy under long-term clinical exposure. Similarly, manganese-containing nanomedicines may accumulate in the liver and spleen, and short-term efficacy or safety findings in ApoE-knockout mouse models neither establish a macrophage-specific causal relationship nor represent the long-term biodistribution, organ toxicity, and safety relevant to clinical translation. Most nanomaterial studies currently evaluate only short-term cytotoxicity or several weeks of animal exposure and therefore cannot exclude long-term metal accumulation and chronic immunotoxicity. The Col@HMnO_2_-MM study, in particular, lacks evaluation of long-term manganese toxicity and metal interactions.

## 5 Conclusions

In summary, essential transition metal homeostasis plays a central role in regulating macrophage function and is a key regulatory node in the onset and progression of chronic metabolic diseases such as MASLD, AS, and diabetes. Iron, zinc, copper, cobalt, and manganese modulate macrophage function through multiple mechanisms, including shaping context-dependent macrophage activation states and functions, modulating oxidative stress, and altering inflammatory cytokine release via signaling pathways such as NF-κB, STAT3, and HIF-1α. Metal homeostasis imbalance-induced macrophage dysfunction is increasingly recognized as a contributor to disease progression by promoting inflammatory responses, disrupting lipid metabolism, impairing insulin sensitivity, and damaging target tissues like liver, blood vessels, pancreas and adipose tissue. To systematically illustrate the regulatory interplay between transition metal imbalance and macrophage dysfunction in chronic metabolic diseases, a schematic overview of this multi-targeted network is presented in **Figure 3**.

**Figure 3:**
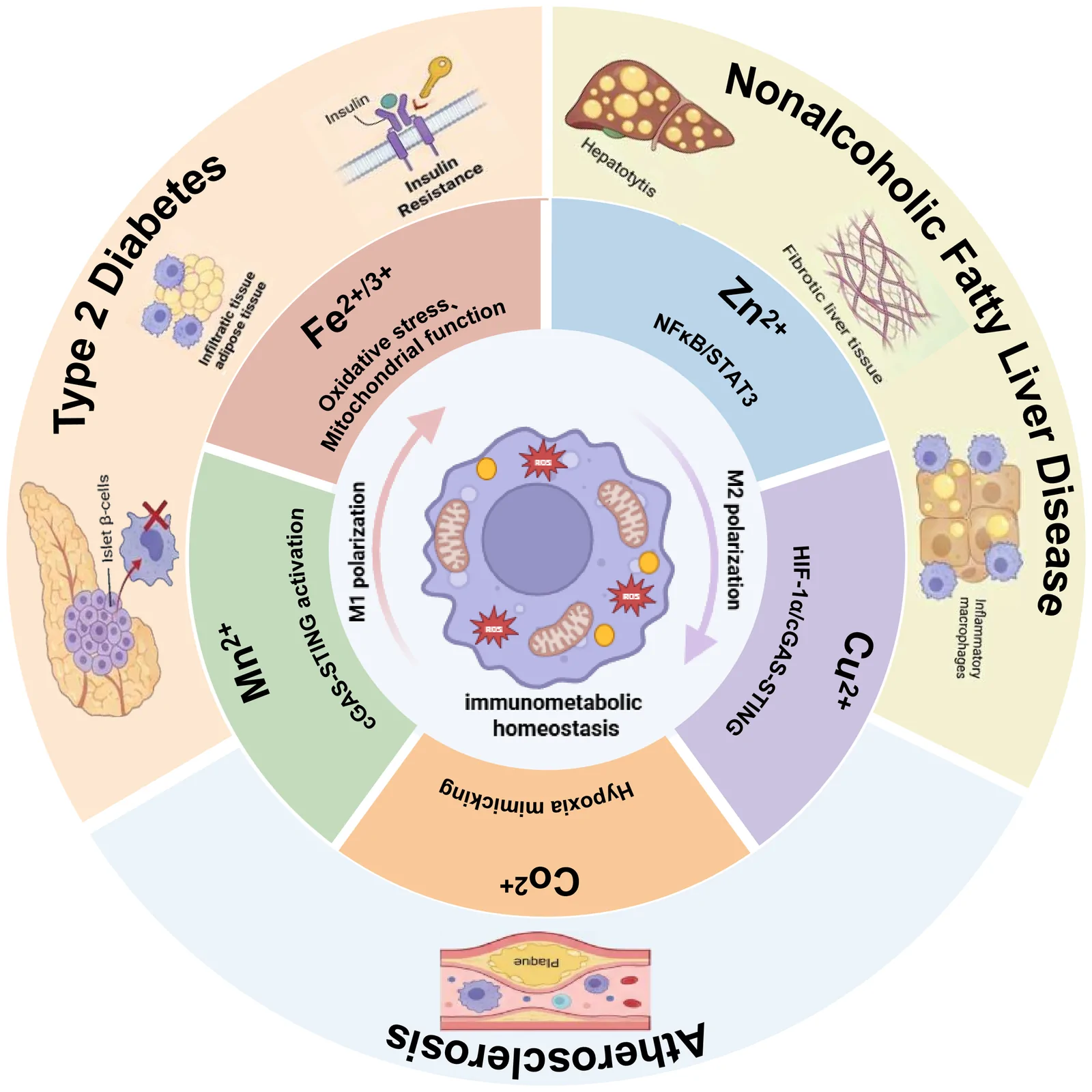
Transition metal imbalance disrupts macrophage immunometabolic homeostasis and drives the pathogenesis of chronic metabolic diseases. Schematic was created with [BioRender.com] (https://BioRender.com).

Iron overload is associated with M2 polarization in macrophages but may also promote foam cell formation and β-cell damage; iron deficiency is associated with M1 polarization and may exacerbate inflammation. Copper and CuONPs activate pro-inflammatory signaling in macrophages, may accelerate AS progression. Zinc deficiency impairs macrophage function and worsens insulin resistance, while appropriate zinc supplementation suppresses pro-inflammatory responses. Cobalt induces macrophage cytotoxicity and inhibits migration *in vitro* via ROS, and manganese exhibits context-dependent regulation of macrophage polarization with therapeutic potential in inflammatory diseases.

Current studies have laid the foundation for understanding the molecular crosstalk between metal homeostasis and macrophage dysfunction, but several limitations remain, including incomplete characterization of signaling pathways, lack of long-term exposure data for metal nanoparticles, and insufficient understanding of macrophage subpopulations dynamics in different disease stages. Future research should focus on clarifying the molecular mechanisms of metal-regulated macrophage subpopulations function, exploring the temporal dynamics and dose thresholds of metal effects, and developing targeted metal-homeostasis-based therapeutic strategies to **modulate** macrophage dysfunction. Integrating clinical cohort studies to analyze the relationships among metal levels, macrophage dysfunction markers, and disease stages will also facilitate the translation of basic research into clinical applications, providing new avenues for the prevention and treatment of chronic metabolic diseases. These metal–macrophage immunometabolic feedback loops help explain why metal dyshomeostasis and macrophage dysfunction can persist and accelerate chronic metabolic disease progression. They also suggest that metal-based therapies should be designed to interrupt specific feedback loops rather than simply correct metal levels. Future studies should define the temporal and dose-dependent dynamics of these loops to optimize chelation, supplementation, or nanoparticle-based interventions.
